# Active-metal template clipping synthesis of novel [2]rotaxanes

**DOI:** 10.3762/bjoc.19.130

**Published:** 2023-11-20

**Authors:** Cătălin C Anghel, Teodor A Cucuiet, Niculina D Hădade, Ion Grosu

**Affiliations:** 1 Babeș-Bolyai University, Faculty of Chemistry and Chemical Engineering, Supramolecular Organic and Organometallic Chemistry Centre, 11 Arany Janos Str., RO-400028-Cluj-Napoca, Romania; 2 University of Bucharest, Faculty of Chemistry; Department of Organic Chemistry, Biochemistry and Catalysis, Research Centre of Applied Organic Chemistry, 90-92 Panduri Street, RO-050663 Bucharest, Romania

**Keywords:** active-metal template, clipping, copper(I)-catalyzed alkyne–azide cycloaddition, mechanically interlocked structures, [2]rotaxanes

## Abstract

Mechanically interlocked molecules (MIMs) have been important synthetic targets in supramolecular chemistry due to their beautiful structures and intriguing properties. We present herein a new synthetic strategy to access [2]rotaxanes, namely active-metal template clipping. We discuss the design of the target [2]rotaxanes, synthesis and characterization of the axle, macrocycle precursors and macrocycles as well as preparation of the final [2]rotaxanes by active template copper(I)-catalyzed alkyne–azide cycloaddition (CuAAC) as key step of the synthesis. HRMS and NMR experiments have been performed to confirm the formation of the interlocked structures.

## Introduction

**Figure 1:**
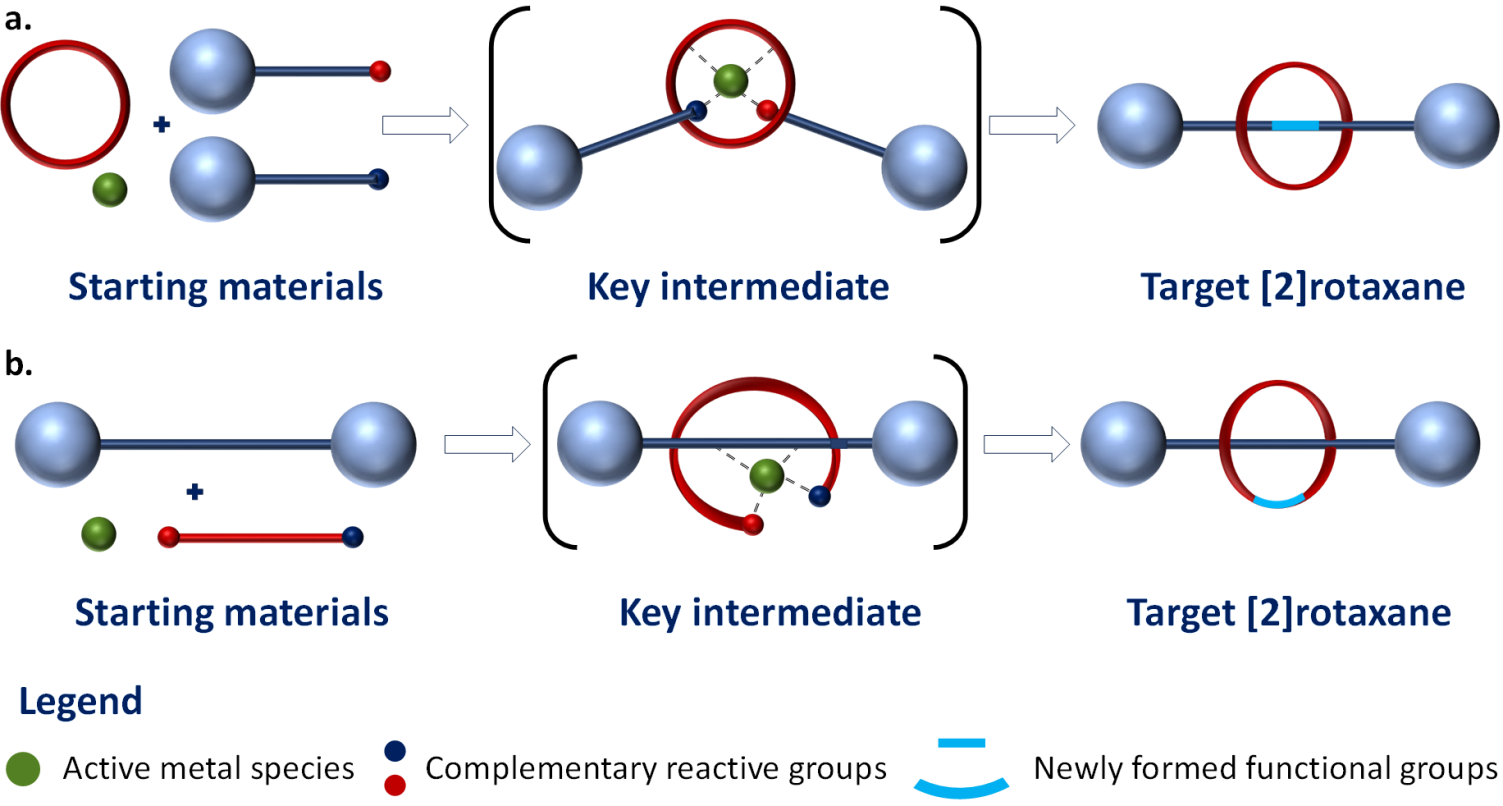
a. Active-metal template (reported in the literature) and b. active-metal template clipping (used in this work) methods.

Since its birth, in the late sixties [1–3], the field of mechanically interlocked molecules (MIMs), including rotaxanes, has gained significant attention culminating by recognition with a Nobel prize in Chemistry in 2016. While initially obtained as chemical curiosities, rotaxanes offer now exciting opportunities for scientific advancements in supramolecular chemistry and applications in various fields ranging from molecular machines and switches [4–9] to catalysis [10–11], molecular electronics [12], and drug delivery [13]. In spite of the important advancements in this field, the synthesis and characterization of these fascinating, complex molecules remains an important challenge and often requires intricate synthetic approaches. After the first templated synthesis of a [2]catenane, reported by Sauvage in 1983 [14], the synthetic strategies employed to access MIMs, including catenanes, rotaxanes and molecular knots, have grown in number and complexity of the obtained structures [15]. Thus, currently, synthesis of rotaxanes is usually performed through clipping, capping, snaping or active-metal template strategies [12–23]. Other known methods are shrinking [24–25], swelling [26] or hydrogen-bond-mediated transition state stabilization [27–29], the latter resembling active-metal template synthesis. Regardless of the method used, the presence of supramolecular interactions that pre-organize rotaxane’s components is crucial for an efficient synthesis. The precursors of rotaxanes are supramolecular architectures held together by numerous interactions leading to diverse motifs such as ammonium crown ether (ion-dipole, hydrogen bonding) [30–31], metal-ion template (coordination bonds [22,32], ion-dipol [16], donor–acceptor (charge transfer, π–π stacking) [30,33], and oligoamide macrocycle-hydrogen acceptors (hydrogen bonding) [20,34]. In active-metal template methods (Figure 1) the metal ion acts both as template and catalyst for the reaction used to complete rotaxane synthesis, hence the name “active template”. This strategy was first applied by Saito and co-workers [35], using a Cu(I) template to catalyze a Glaser coupling reaction. The method has been extended for other reactions, for example Ni-catalyzed homocoupling of primary alkyl bromides [36] and cooper(I)-catalyzed alkyne–azide cycloaddition (CuAAC) click chemistry [37]. In all these cases a templated metal ion–macrocycle complex is used to catalyze the rotaxane formation by connecting two components of the dumbbell-shaped molecule (Figure 1a).

In this context, we envisaged to combine active-metal template and clipping strategies and we report herein the synthesis and characterization of novel [2]rotaxanes by this new method that we name active-metal template clipping (Figure 1b). Thus, our approach involves the use of a metal ion connected to the dumbbell-shaped molecule which catalyzes the macrocyclization reaction around the axle (Figure 1b).

## Results and Discussion

In order to access the target [2]rotaxanes we made use of the CuAAC reaction, performed in the presence of a copper(I) *N*-heterocyclic carbene, a very stable and efficient class of catalysts used in CuAAC click chemistry [38–40]. The choice of CuAAC as key step to accomplish the synthesis of the [2]rotaxanes was motivated by its high efficiency [41]. In addition, this reaction proved its versatility for rotaxanes synthesis [15], including chiral [2]rotaxanes [42].

**Figure 2:**
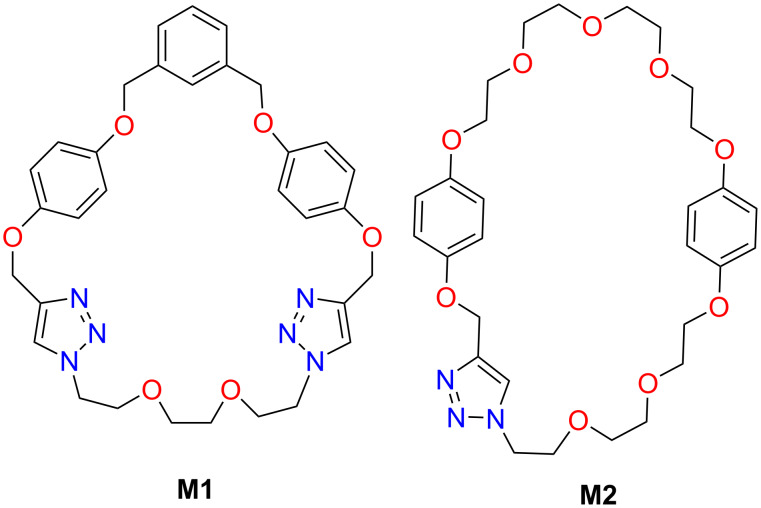
Macrocyclic components of the [2]rotaxanes.

**Scheme 1:**
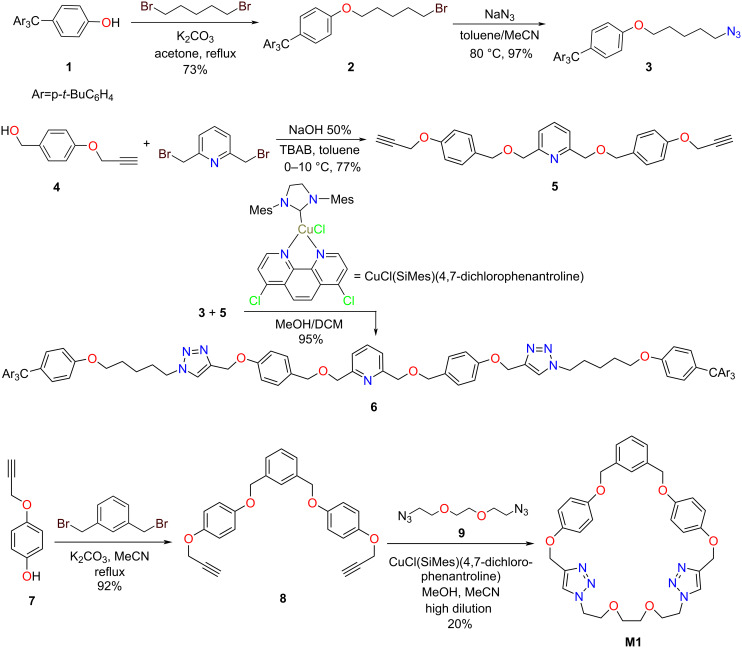
Synthesis of the key intermediates **6** and **8** and of the reference macrocycle **M1**.

The macrocycles **M1** and **M2** (Figure 2) were designed to be obtained from precursors containing two 1,4-dioxobenzene fragments required to facilitate π–π interactions with the pyridine unit in the axle (compound **6** in Scheme 1) and confer stability to the supramolecular complex necessary for the formation of [2]rotaxanes.

For the synthesis of the axle unit (compound **6** in Scheme 1) we considered a convergent synthetic approach that consist in preparation of the stopper, functionalized with azide groups, decoration of the central pyridine-based moiety with propargyl groups and their connection by CuAAC click chemistry. Thus, as stoppers we have employed a tetraarylmethane bulky fragment **1** (Scheme 1), widely used in the synthesis of rotaxanes because of its symmetry which results in simpler NMR spectra [43]. Compound **1** was reacted with an excess of 1,5-dibromopentane to obtain compound **2** that was further transformed into the azide-functonalized stopper **3** after substitution of bromine with azide. The dialkyne-decorated pyridine **5** was prepared starting from 2,6-bis(bromomethyl)pyridine that was reacted with compound **4**, under phase transfer catalysis (Scheme 1).

Finally, the axle **6**, as well as the reference macrocycles **M1** and **M2** [44], were obtained by CuAAC in the presence of CuCl(SIMes)(4,7-diclorophenantroline) as catalyst, with very good yields.

**Scheme 2:**
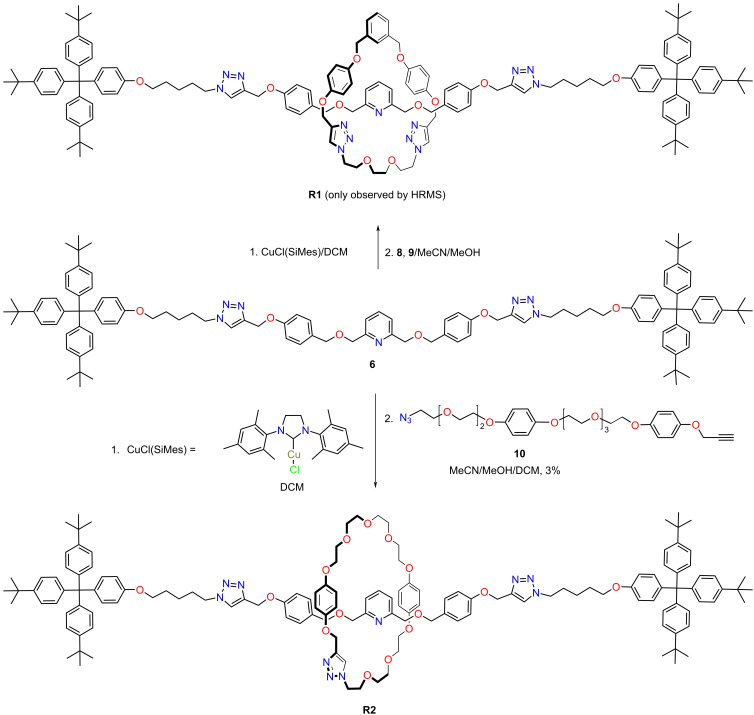
Synthesis of [2]rotaxanes **R1** and **R2**.

**Figure 3:**
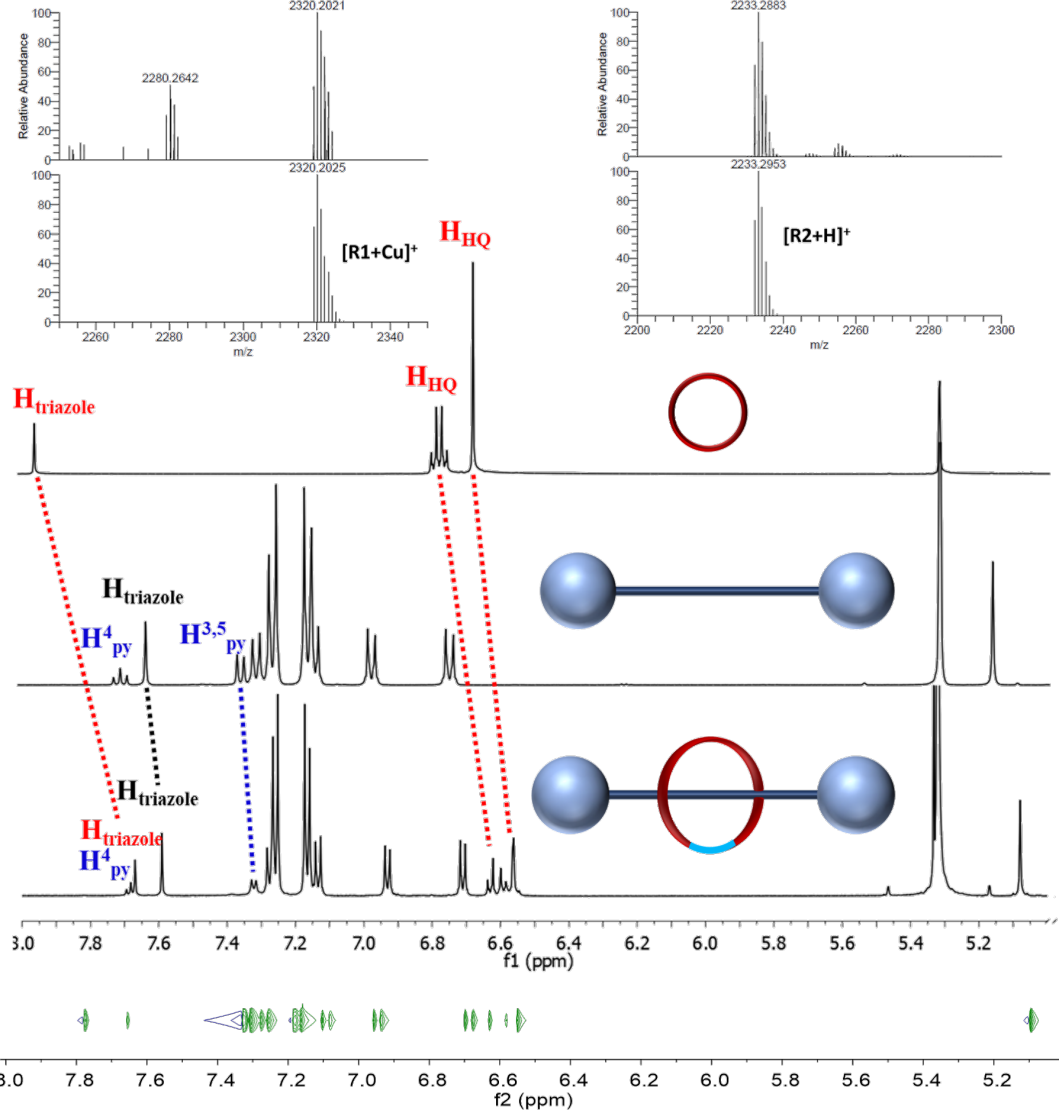
Top: HRESI(+)-MS spectrum of the rotaxane **R1** (left) and **R2** (right) [experimental (top) and calculated (bottom) isotopic patterns]; middle: comparative ^1^H NMR spectra of the aromatic region of rotaxane **R2** with the macrocycle and with the axle (CD_2_Cl_2_, 400 MHz); bottom: fragment of ^1^H-DOSY NMR (MeCN-*d*_3_, 400 MHz) spectrum of compound **R2**.

Next, we set to study the ability of compound **6** to form copper(I) complexes able to act as active-metal templates for [2]rotaxane synthesis. Therefore, complexation studies of **6** with CuCl(SIMes) were performed and analyzed by HRMS, demonstrating formation of the complex (see Supporting Information File 1, Figure S15). The HRESI(+)-MS spectrum showed the base peak at *m*/*z* = 2029.1594 corresponding to [Cu(**6**)(SIMes)]^+^. With this complex in our hands we set to investigate the synthesis of [2]rotaxanes using both a [1 + 1] macrocyclization clipping reaction to obtain **R1** and an intramolecular macrocyclization to access **R2** (Scheme 2). In both cases formation of the [2]rotaxanes was observed by HRESI(+)-MS (Figure 3, top). Thus, the HRESI(+)-MS spectrum of **R1** showed two characteristic peaks at *m*/*z* = 2280.2642 corresponding to the [**R1** + Na]^+^ adduct and at *m*/*z* = 2320.2021 assigned to the [**R1**+Cu]^+^ ion (Figure 1, top, left and Figure S17 in Supporting Information File 1), while the HRESI(+)-MS of **R2** displayed the base peak at *m*/*z* = 2233.2883 corresponding to the protonated molecular ion. Formation of the [2]rotaxanes was further confirmed by MS^2^ experiments, i.e., isolation of the peaks corresponding to the [2]rotaxanes followed by collision-induced dissociation (CID) yielded the protonated molecular ions of the axle **6** and macrocycles **M1** and **M2**, respectively (Figures S18 and S28 in Supporting Information File 1).

However, only [2]rotaxane **R2** could be isolated as pure compound in 3% yield, after several purification operations by column chromatography. The improved results obtained in the intramolecular macrocyclization for the clipping reaction as compared to the [1 + 1] strategy are in line with the yields displayed by the two strategies for the preparation of reference macrocycles **M1** (20%) and **M2** (44%) [44].

The ^1^H NMR spectrum of [2]rotaxane **R2** displayed signals corresponding to both axle and macrocycle in a 1:1 ratio (see Supporting Information File 1, Figure S19 for the full NMR spectrum). Thanks to the symmetry of the axle and non-symmetry of the macrocycle the signals of the components could be easily distinguished by integrals. The aliphatic region is crowded with triplets corresponding to the ethyleneglycole and pentyl linkers. However, the difference in symmetry allowed the differentiation of the triplets at δ = 4.18 ppm and δ = 3.78 ppm corresponding to CH_2_ protons from the pentyl linker, also confirmed by H,H-COSY NMR. Comparison of the ^1^H NMR spectra of **R2** and its components **6** and **M2** revealed substantial differences in the chemical shifts of different signals of **R1** and **6**, **M2**, respectively. In Figure 3 (middle) is presented the zoomed aromatic region indicating a small shielding of all the axle protons (e.g., the signals corresponding to H_3,5_ and H_4_ pyridine protons are shifted from δ = 7.35 ppm to δ = 7.32 ppm and from δ = 7.70 ppm to δ = 7.68 ppm, respectively).

The signals of the macrocycles are more shifted, with the triazole proton presenting an upfield shift from δ = 7.95 ppm to δ = 7.65 ppm and the hydroquinone protons are upfield shifted from δ = 6.77/6.67 ppm to δ = 6.60/6.58 ppm.

In order to validate formation of **R1** we also recorded a room-temperature H,H-ROESY NMR spectrum (Supporting Information File 1, Figure S23). Surprisingly, no correlation between signals corresponding to the axle and macrocycle have been observed. This result could be explained by rapid movement of the macrocycle along the axle with no preferred position. However, formation of the rotaxane could be validated by ^1^H-DOSY NMR experiments (Figure 3, and Supporting Information File 1, Figure S26) indicating that the macrocycle and the axle diffuse as a whole with a diffusion coefficient of 4.2 × 10^−10^ m^2^/s.

## Conclusion

In summary, we introduced herein a new synthetic strategy of [2]rotaxanes that combined active-metal template and clipping methods to yield the target interlocked molecules via [1 + 1] or intramolecular macrocyclizations, realized through CuAAC reactions catalyzed by axle-copper(I) *N*-heterocyclic carbene complexes. The [2]rotaxane obtained by [1 + 1] clipping (**R1**) could be only observed by HRMS, most probably because of the lower efficiency of the [1 + 1] reaction combined with the smaller size of the macrocycle. The [2]rotaxane **R2** obtained by intramolecular clipping was successfully isolated as unique compound and its structure was validated by HRMS, ^1^H,^13^C and ^1^H-DOSY NMR experiments. Our results proved that the CuAAC reaction catalyzed by copper(I) *N*-heterocyclic carbenes can be successfully used in the synthesis of [2]rotaxanes. This, combined with the fast and simple assembly of [CuCl(SIMes)] [45–46] could lead to the development of a Cu(I) NHC click chemistry in the field of MIMs. This approach is a proof of concept which will be further developed along with improvements in the design of the supramolecular interactions between the axle station(s) and macrocycle components in order to provide an important alternative method in rotaxane synthesis.

## Experimental

### General experimental data

Commercially available reagents were used without further purification. Compounds **1** [43,47], **4** [48], **9** [49], **10** [44] and **M2** [44] were synthesized according to procedures reported in the literature. Thin-layer chromatography (TLC) was performed on silica gel 60 coated aluminium F_254_ plates and visualized by UV irradiation at 254 nm. Preparative column chromatography was carried out using silica gel 60 (0.040–0.063 mm) from Merck. The NMR spectra were recorded on Bruker Avance 400 MHz or Bruker Avance 600 MHz spectrometers. Chemical shifts (δ) are reported in parts per million (ppm) relative to the residual solvent peak. High-resolution mass spectra were recorded on a ThermoScientific (LTQ XL, Orbitrap) spectrometer, in positive ion mode, using Electrospray or APCI techniques.

### Experimental procedures and characterization of compounds

**Compound 2:** In a round-bottom flask compound **1** (1.52 g, 3.02 mmol, 1 equiv) was dissolved in acetone (100 mL), followed by the addition of K_2_CO_3_ (4.17 g, 30.2 mmol, 10 equiv). After 30 min of stirring 1,5-dibromopentane (3.47 g, 15.1 mmol, 5 equiv) was added and the mixture was kept under reflux for 48 hours. Upon cooling, the compound was isolated after filtration and solvent evaporation. The compound was purified by column chromatography (silica, toluene/petroleum ether = 1:4) to afford a white solid (1.44 g, 73%); mp 222–225 °C, *R*_f_ = 0.44 (silica, toluene/petroleum ether = 1:4). ^1^H NMR (CDCl_3_, 600 MHz) δ 7.23 (d, ^3^*J* = 8.6 Hz, 6H, H_Ar_), 7.10–7.04 (overlapped signals, 8H, H_Ar_), 6.75 (d, ^3^*J* = 8.9 Hz, 2H, H_Ar_), 3.94 (t, ^3^*J* = 6.3 Hz, 2H, OC*H*_2_), 3.43 (t, ^3^*J* = 6.8 Hz, 2H, C*H*_2_Br), 1.93 (qv, ^3^*J* = 6.9 Hz, 2H, C*H*_2_), 1.83–1.77 (m, 2H, C*H*_2_), 1.65–1.58 (m, 2H, C*H*_2_), 1.30 (s, 27H, C(C*H*_3_)_3_) ppm; ^13^C NMR (CDCl_3_, 150 MHz) δ 156.9, 148.5, 144.3, 139.7, 132.4, 130.9, 124.2, 113.1, 67.5, 63.2, 34.3, 33.8, 32.7, 31.5, 28.7, 25.1 ppm; HRAPCI(+)–MS (*m*/*z*): [M − C_11_H_14_BrO]^+^ calcd for C_31_H_39_: 411.3046; found, 411.3022; [M − C_10_H_13_]^+^ calcd for C_32_H_40_BrO: 521.2237; found, 521.2233

**Compound 3, adapted procedure** [50]: Compound **2** (2.35 g, 3.60 mmol, 1 equiv) and NaN_3_ (1.40 g, 21.56 mmol, 6 equiv) were dissolved in MeCN (100 mL) and toluene (50 mL) in a round-bottom flask. The mixture was refluxed until reaction completion as monitored by TLC. Upon cooling, water was added (100 mL) and the compound was extracted with DCM (3 × 75 mL). The combined organic phases were washed with water and brine, followed by drying over MgSO_4_. After solvent removal the compound was obtained as a white solid (2.14 g, 97%). ^1^H NMR (CDCl_3_, 600 MHz) δ 7.23 (d, ^3^*J* = 8.5 Hz, 6H, H_Ar_), 7.10–7.04 (overlapped signals, 8H, H_Ar_), 6.75 (d, ^3^*J* = 8.8 Hz, 2H, H_Ar_), 3.94 (t, ^3^*J* = 6.2 Hz, 2H, OC*H*_2_), 3.30 (t, ^3^*J* = 6.8 Hz, 2H, C*H*_2_N_3_), 1.80 (qv, ^3^*J* = 6.5 Hz, 2H, C*H*_2_), 1.68 (qv, ^3^*J* = 7.0 Hz, 2H, C*H*_2_), 1.59–1.55 (m, 2H, C*H*_2_), 1.30 (s, 27H, C(C*H*_3_)_3_) ppm.

**Compound 5:** Compound **4** (0.76 g, 4.69 mmol, 2.2 equiv) was dissolved in toluene (50 mL) and the solution was cooled at 0 °C on an ice bath. NaOH 50% (2.25 g, 56.28 mmol, 26.4 equiv) was added dropwise, followed by TBAB (0.17 g, 0.53 mmol, 0.25 equiv). 2,6-Bis(bromomethyl)pyridine (0.56 g, 2.11 mmol, 1 equiv) was added after 30 minutes and the mixture was stirred for 2 days at room temperature. After solvent evaporation, water (50 mL) was added and the compound was extracted with DCM (3 × 50 mL). The combined organic phases were washed with water and brine, followed by drying over MgSO_4_. After solvent evaporation the compound was purified by column chromatography (silica, acetone/toluene = 1:6, *R*_f_ = 0.47) to afford a yellowish solid (0.69 g, 77%); mp 128–130 °C, *R*_f_ = 0.47 (silica, acetone/toluene = 1:6). ^1^H NMR (CD_2_Cl_2_, 600 MHz) δ 7.72 (t, ^3^*J* = 7.7 Hz, 1H, H_pyridine_), 7.36 (d, ^3^*J* = 7.7 Hz, 2H, H_pyridine_), 7.33 (d, ^3^*J* = 8.6 Hz, 4H, H_Ar_), 6.96 (d, ^3^*J* = 8.6 Hz, 4H, H_Ar_), 4.70 (d, ^3^*J* = 2.4 Hz, 4H, C*H*_2_ (propargyl)), 4.60 (s, 4H, C*H*_2_), 4.55 (s, 4H, C*H*_2_), 2.57 (t, ^3^*J* = 2.4 Hz, 2H, C*H*) ppm; ^13^C APT NMR (CD_2_Cl_2_, 150 MHz) δ 158.6, 157.8, 137.7, 132.1, 129.9, 120.5, 115.4, 79.2, 75.8, 73.6, 72.9, 56.5.

**Compound 6:** Compound **5** (0.36 g, 0.6 mmol, 1 equiv) and compound **3** (0.82 g, 1.33 mmol, 2.2 equiv) were dissolved in DCM/MeOH (20 mL/5 mL). CuCl(SIMes)(4,7-dichlorophenantroline) (39 mg, 0.06 mmol, 0.1 equiv) was added and the mixture was stirred for 4 days at room temperature. The solvent was evaporated and the residue was dissolved in DCM (25 mL), washed with water and brine. After drying over MgSO_4_ the solvent was evaporated. The compound was purified on column chromatography (silica, acetone/toluene = 1:3, *R*_f_ = 0.47) to afford a white solid (1.05 g, 95%); mp 180–181 °C, *R*_f_ = 0.47 (silica, acetone/toluene = 1:3). ^1^H NMR (CD_2_Cl_2_, 400 MHz) δ 7.70 (t, ^3^*J* = 7.7 Hz, 1H, H_pyridine_), 7.63 (s, 2H, H_triazole_), 7.35 (d, ^3^*J* = 7.7 Hz, 2H, H_pyridine_), 7.31 (d, ^3^*J* = 8.6 Hz, 4H, H_Ar_), 7.26 (d, ^3^*J* = 8.6 Hz, 4H, H_stopper_), 7.18–7.10 (overlapped signals, 12H, H_stopper_), 6.97 (d, ^3^*J* = 8.6 Hz, 4H, H_Ar_), 6.74 (d, ^3^*J* = 8.9 Hz, 4H, H_stopper_), 5.17 (s, 4H, C*H*_2_), 4.60 (s, 4H, C*H*_2_), 4.54 (s, 4H, C*H*_2_), 4.37 (t, 4H, ^3^*J* = 7.2 Hz, NC*H*_2_), 3.92 (t, ^3^*J* = 6.2 Hz, 4H, OC*H*_2_), 1.97 (qv, ^3^*J* = 7.5 Hz, 4H, C*H*_2_), 1.83–1.77 (m, 4H, C*H*_2_), 1.54–1.44 (m, 4H, C*H*_2_), 1.30 (s, 54H, C(C*H*_3_)_3_) ppm; ^13^C APT NMR (CD_2_Cl_2_, 100 MHz) δ 158.6, 157.4, 148.9, 145.1, 144.4, 140.2, 137.6, 132.4, 131.5, 131.0, 130.0, 124.8, 123.3, 120.5, 115.2, 113.7, 73.6, 73.0, 67.9, 63.7, 62.6, 50.8, 34.8, 31.6, 30.6, 29.2, 23.8 ppm; HRESI(+)-MS (*m*/*z*): [M + Na]^+^ calcd for C_111_H_131_N_7_O_6_Na: 1682.0087; found, 1682.0065.

**Compound 8: ***m*-Xylylene dibromide (1.25 g, 5.20 mmol, 1 equiv), compound **7** (1.77 g, 11.96 mmol, 2.3 equiv) and K_2_CO_3_ (7.18 g, 52 mmol, 10 equiv) were dissolved in MeCN (80 mL). The reaction mixture was refluxed overnight. After cooling down, the mixture was filtered and the solvent was evaporated. The residue was dissolved in AcOEt (50 mL) and washed with aqueous 10% NaOH (3 × 25 mL). The organic phases were dried on MgSO_4_ and the solvent removed under vacuum to give a white solid (1.90 g, 92%); mp 72–73 °C, *R*_f_ = 0.47 (silica, ethyl acetate/petroleum ether = 1:3);^1^H NMR (CDCl_3_, 600 MHz) δ 7.49 (s, 1H, H_Ar_), 7.42–7.36 (overlapped signals, 3H, H_Ar_), 6.93 (s, 8H, H_Ar_), 5.03 (s, 4H, CH_2_), 4.65 (d, 4H, ^4^*J*= 2.3 Hz, C*H*_2_(propargyl)), 2.52 (t, ^4^*J* = 2.3 Hz, C*H*_2_(propargyl)) ppm; ^13^C NMR (CDCl_3_, 150 MHz) δ 153.7, 152.0, 137.7, 129.0, 127.1, 126.6, 116.2, 115.9, 79.0, 75.5, 70.6, 56.7 ppm.

**Macrocycle M1:** CuCl(SIMes)(4,7-dichlorophenantroline) (23 mg, 0.036 mmol, 0.1 equiv) was dissolved in MeOH (50 mL). 1,2-Bis(2-azidoethoxy)ethane (72 mg, 0.36 mmol, 1 equiv) and **8** (143 mg, 0.36 mmol, 1 equiv) dissolved in MeCN (5 mL) were added dropwise during 12 hours by using a push-syringe. The mixture was stirred for 48 hours at room temperature. The solvent was evaporated and the residue was dissolved in DCM (25 mL), followed by washings with *N*-(2-hydroxyethyl)ethylenediamine-*N*,*N*′,*N*′-triacetic acid (HEEDTA) saturated solution (3 × 20 mL), water and brine. After drying on MgSO_4_ the solvent was evaporated. The compound was purified by column chromatography (silica, acetone/methanol = 20:1, *R*_f_ = 0.33) to afford a white solid (45 mg, 20%). *R*_f_ = 0.33 (silica, acetone/methanol = 20:1 v/v). ^1^H NMR (CDCl_3_, 400 MHz) δ 7.73 (s, 2H, H_Ar_), 7.32 (t, ^3^*J* = 7.8 Hz, 1H, H_Ar_), 7.28 (s, 1H, H_Ar_), 7.22 (d, ^3^*J* = 7.8 Hz, 2H, H_Ar_), 5.13 (s, 4H, OC*H*_2_), 5.08 (s, 4H, OC*H*_2_), 4.48 (t, ^3^*J* = 5.1 Hz, 4H, NC*H*_2_), 3.79 (t, ^3^*J* = 5.1 Hz, 4H, OC*H*_2_), 3.51 (s, 4H, OC*H*_2_) ppm; ^13^C APT NMR (CDCl_3_, 100 MHz) δ 152.5, 144.4, 138.0, 129.0, 126.4, 126.1, 124.0, 116.9, 115.8, 70.6, 70.4, 69.4, 62.6, 50.3 ppm; HRESI(+)-MS (*m*/*z*): [M + Na]^+^ calcd for C_32_H_34_N_6_O_6_Na: 621.2432; found, 621.2419.

### General procedure for the synthesis of the [2]rotaxanes

The dumbbell-shaped compound (0.045 mmol, 1 equiv) and CuCl(SiMes) (0.045 mmol, 1 equiv) were dissolved in DCM (20 mL), the mixture was stirred for 1 hour and the solvent was evaporated. The residue and the macrocycle precursor(s) (0.1125 mmol, 2.5 equiv) were dissolved in a mixture of MeCN/MeOH/DCM (8 mL/8 mL/12 mL) and stirred for 6 days. The solvent was evaporated, the residue was dissolved in AcOEt (25 mL), followed by washings with HEEDTA saturated solution (3 × 20 mL), water and brine. After drying over MgSO_4_ the solvent was evaporated and the resulting residue was analyzed by HRMS and NMR.

**Rotaxane R1**: HRESI(+)-MS (*m*/*z*): [M + Na]^+^ calcd for C_143_H_165_N_13_O_12_Na: 2280.2626; found, 2280.2642; [M + Cu]^+^ calcd for C_143_H_165_N_13_O_12_Cu: 2320.2025; found, 2320.2021.

**[2]Rotaxane R2**: The compound was purified by column cromatography (silica, solvent gradient from acetone/toluene = 1:3 to acetone/toluene = 1:2 , *R*_f_ = 0.33) to afford a white solid (3 mg, 3%); *R*_f axle_ = 0.7, *R*_f macrocycle_ = 0.18 (silica, acetone/toluene = 1:2); ^1^H NMR (CD_2_Cl_2_, 600 MHz) δ 7.71–7.66 (overlapped signals, 2H, H_pyridine,_ H_triazole_), 7.59 (s, 2H, H_triazole_), 7.32 (d, ^3^*J* = 7.7 Hz, 2H, H_Ar_), 7.30–7.24 (overlapped signals, 16H, H_stopper_, H_Ar_), 7.17 (d, ^3^*J* = 8.6 Hz, 12H, H_Ar_), 7.13 (d, ^3^*J* = 8.8 Hz, 4H, H_Ar_), 6.93 (d, ^3^*J* = 8.6 Hz, 4H, H_Ar_), 6.71 (d, ^3^*J* = 8.8 Hz, 4H, H_stopper_), 6.65–6.54 (overlapped signals, 8H, H_Ar macrocycle_), 5.08 (s, 4H, C*H*_2_), 4.86 (s, 2H, C*H*_2_), 4.57 (s, 4H, C*H*_2_), 4.51 (s, 4H, C*H*_2_), 4.38 (t, ^3^*J* = 5.1 Hz, 2H, C*H*_2_), 4.18 (t, ^3^*J* = 7.4 Hz, 4H, C*H*_2_), 3.91–3.85 (overlapped signals, 6H, C*H*_2_), 3.78 (t, ^3^*J* = 6.4 Hz, 4H, C*H*_2_), 3.73 (t, ^3^*J* = 5.1 Hz, 2H, C*H*_2_), 3.70–3.64 (overlapped signals, 6H, C*H*_2_), 3.60–3.51 (overlapped signals, 12H, C*H*_2_), 1.75 (qv, ^3^*J* = 7.6 Hz, 4H, C*H_2_*), 1.64 (qv, ^3^*J* = 7.3 Hz, 4H, C*H_2_*), 1.35–1.28 (overlapped signals, 58H, C*H*_2_, C(C*H*_3_)_3_) ppm; ^13^C NMR (CD_2_Cl_2_, 150 MHz) δ 158.6, 158.57, 157.4, 153.6, 153.4, 153.3, 152.9, 148.9, 145.1, 144.4, 144.1, 140.1, 137.6, 132.4, 131.4, 131.0, 130.0, 124.9, 124.7, 123.7, 120.4, 116.1, 115.8, 115.7, 115.6, 115.1, 113.8, 73.6, 73.0, 71.26, 71.24, 71.22, 71.0, 70.30, 70.27, 70.1, 68.2, 68.21, 68.18, 67.9, 63.7, 62.6, 62.5, 50.68, 50.65, 34.8, 31.7, 30.4, 29.2, 23.7 ppm; HRESI(+)-MS (*m/z*): [M + H]^+^ calcd for C_140_H_171_N_10_O_15_: 2233.2953; found, 2233.2883.

## Supporting Information

File 1 Copies of NMR and HRMS spectra.
