# Supplementary material for: Active-metal template clipping synthesis of novel [2]rotaxanes
Source: Beilstein J Org Chem. 2023 Nov 20;19:1776–84. doi: 10.3762/bjoc.19.130 (PMC10682515; doi:10.3762/bjoc.19.130)
Supplement: File 1 — Copies of NMR and HRMS spectra. [file Beilstein_J_Org_Chem-19-1776-s001.pdf]

BEILSTEIN JOURNAL OF ORGANIC CHEMISTRY

Supporting Information

for

Active-metal template clipping synthesis of novel [2]rotaxanes

Cătălin C. Anghel, Teodor A. Cucuiet, Niculina D. Hădade and Ion Grosu

Beilstein J. Org. Chem. **2023,** *19,* 1776–1784. doi:10.3762/bjoc.19.130

Copies of NMR and HRMS spectra

License and Terms: This is a supporting information file under the terms of the Creative Commons Attribution License (https://creativecommons.org/licenses/by/4.0). Please note that the reuse, redistribution and reproduction in particular requires that the author(s) and source are credited and that individual graphics may be subject to special legal provisions.
The license is subject to the *Beilstein Journal of Organic Chemistry* terms and conditions: (https://www.beilstein-journals.org/bjoc/terms)

Table of contents

Figure S1: ^{1}H NMR ($CDCl_3$, 600 MHz) spectrum of compound **2**.................... S2
Figure S2: ^{13} C APT NMR ($CDCl_3$, 150 MHz) spectrum of compound **2**.................... S2
Figure S3: APCI(+)-HRMS spectrum of compound **2** S3
Figure S4: ^{1}H NMR ($CDCl_3$, 600 MHz) spectrum of compound **3**.................... S3
Figure S5: ^{1}H NMR (CD_2Cl_2, 600 MHz) spectrum of compound **5** S4
Figure S6: ^{13}C APT NMR (CD_2Cl_2, 150 MHz) spectrum of compound **5** S4
Figure S7: ^{1}H NMR (CD_2Cl_2, 400 MHz) spectrum of compound **6** S5
Figure S8: ^{13}C APT NMR (CD_2Cl_2, 100 MHz) spectrum of compound **6** S5
Figure S9: HRESI(+)-MS spectrum of compound **6**.................... S6
Figure S10: ^{1}H NMR ($CDCl_3$, 600 MHz) spectrum of compound **8**.................... S6
Figure S11: ^{13}C APT NMR ($CDCl_3$, 150 MHz) spectrum of compound **8** S7
Figure S12: ^{1}H NMR ($CDCl_3$, 600 MHz) spectrum of compound **M1**.................... S7
Figure S13: ^{13}C APT NMR ($CDCl_3$, 150 MHz) spectrum of compound **M1** S8
Figure S14: HRESI(+)-MS spectrum of compound **M1** S8
Figure S15: HRMS spectrum of the complex between axle **6** and **CuCl(SIMes)** S9
Figure S16: HRMS spectrum of the complex between axle **6** and **CuCl(SIMes**) [experimental (top) and calculated (bottom) isotopic patterns].................... S9
Figure S17: HRESI(+)-MS spectrum of the rotaxane **R1** [experimental (top) and calculated (bottom) isotopic patterns].................... S10
Figure S18: ESI(+) MS^{2} spectrum of rotaxane **R1** S10
Figure S19: ^{1}H NMR (CD_2Cl_2, 600 MHz) spectrum of compound **R2**.................... S11
Figure S20: ^{13}C NMR (CD_2Cl_2, 150 MHz) spectrum of compound **R2**.................... S11
Figure S21: Zoom ^{1}H NMR (CD_2Cl_2, 600 MHz) spectrum of compound **R2** S12
Figure S22: H,H-COSY NMR (CD_2Cl_2, 400 MHz) spectrum of compound **R2**: aromatic region....................S12
Figure S23: H,H-ROESY NMR (CD_2Cl_2, 400 MHz) spectrum of compound **R2**: aromatic region S13
Figure S24: HSQC NMR (CD_2Cl_2, 400 MHz) spectrum of compound **R2**: aromatic region S13
Figure S25: HMBC NMR (CD_2Cl_2, 400 MHz) spectrum of compound **R2**: aromatic region.................... S14
Figure S26: ^{1}H-DOSY NMR (MeCN-d_3, 400 MHz) spectrum of compound **R2**.................... S14
Figure S27: HRESI(+)-MS spectrum of compound **R2** S15
Figure S28: ESI(+)-MS^{2} spectrum of rotaxane **R2** S15

Copies of NMR and mass spectra

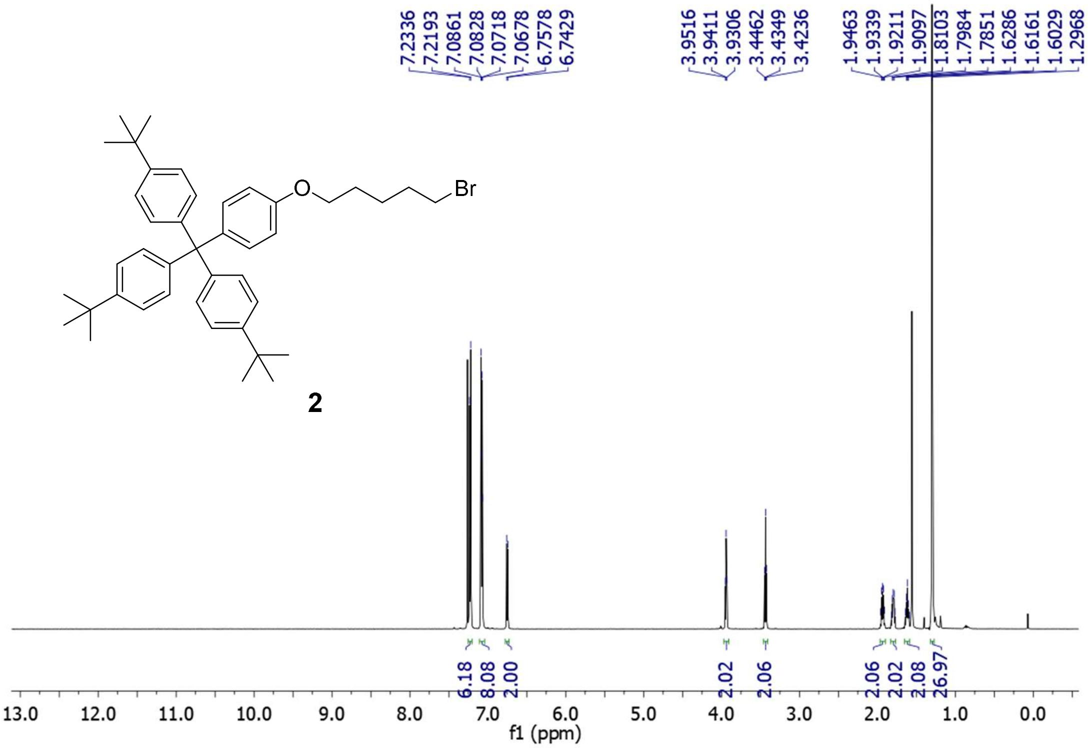

Figure S1: ^{1}H NMR ($CDCl_3$, 600 MHz) spectrum of compound **2**.

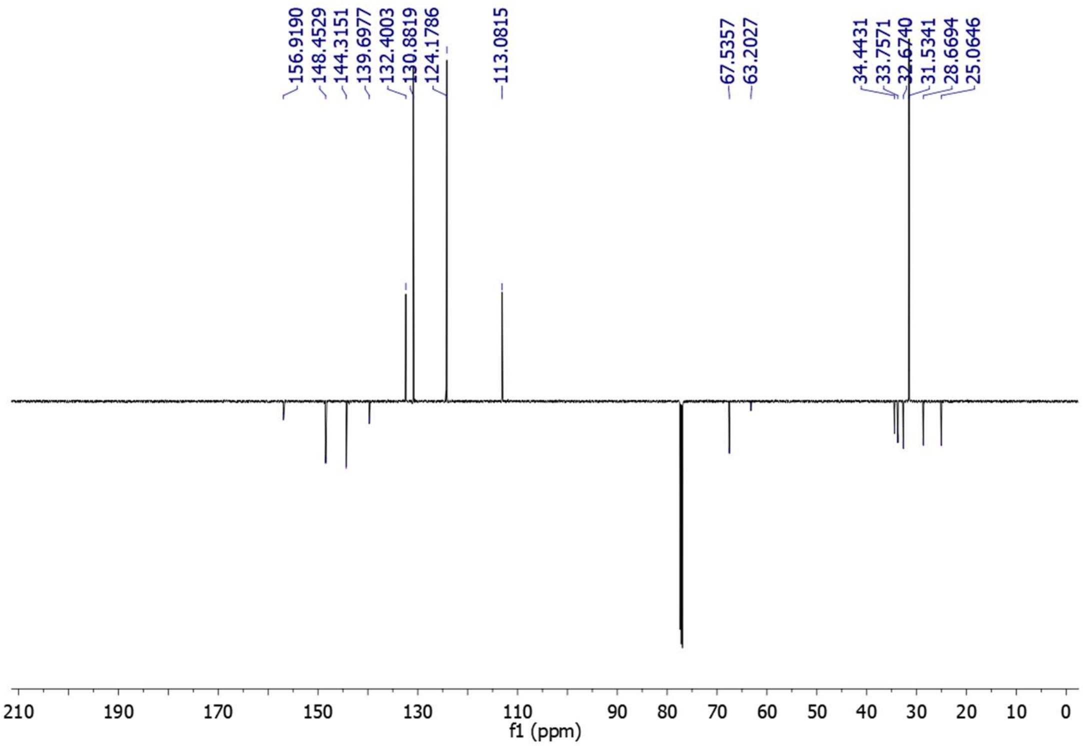

Figure S2: ^{13}C APT NMR ($CDCl_3$, 150 MHz) spectrum of compound **2**.

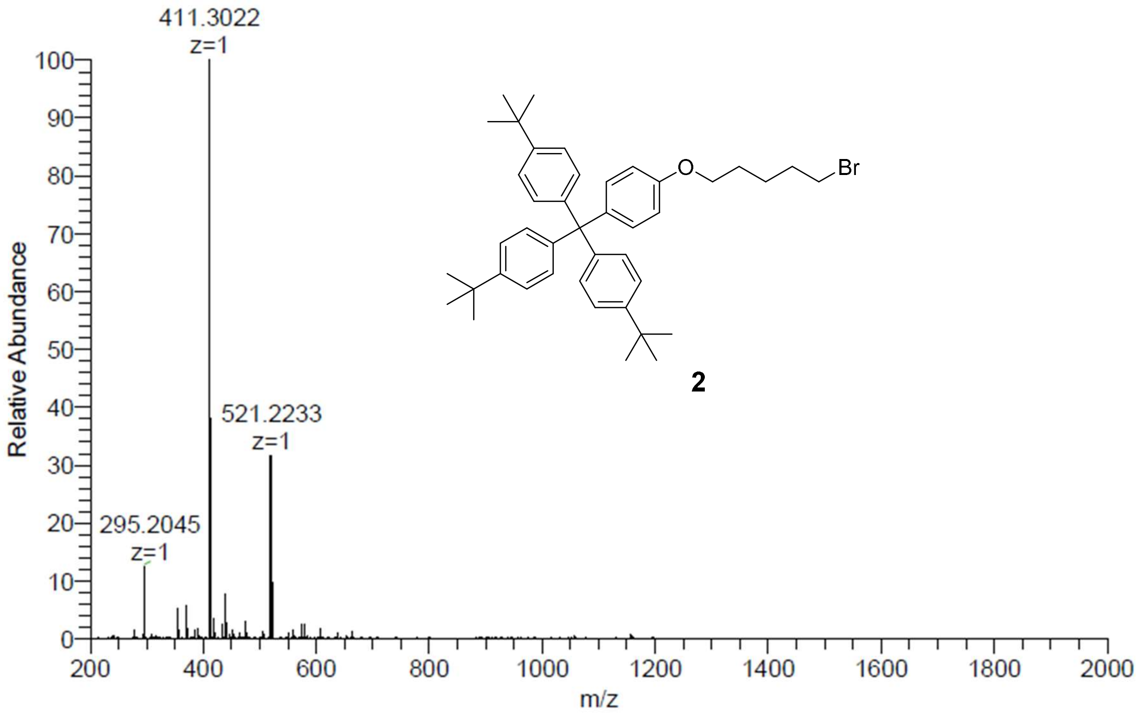

Figure S3: APCI(+)-HRMS spectrum of compound **2**.

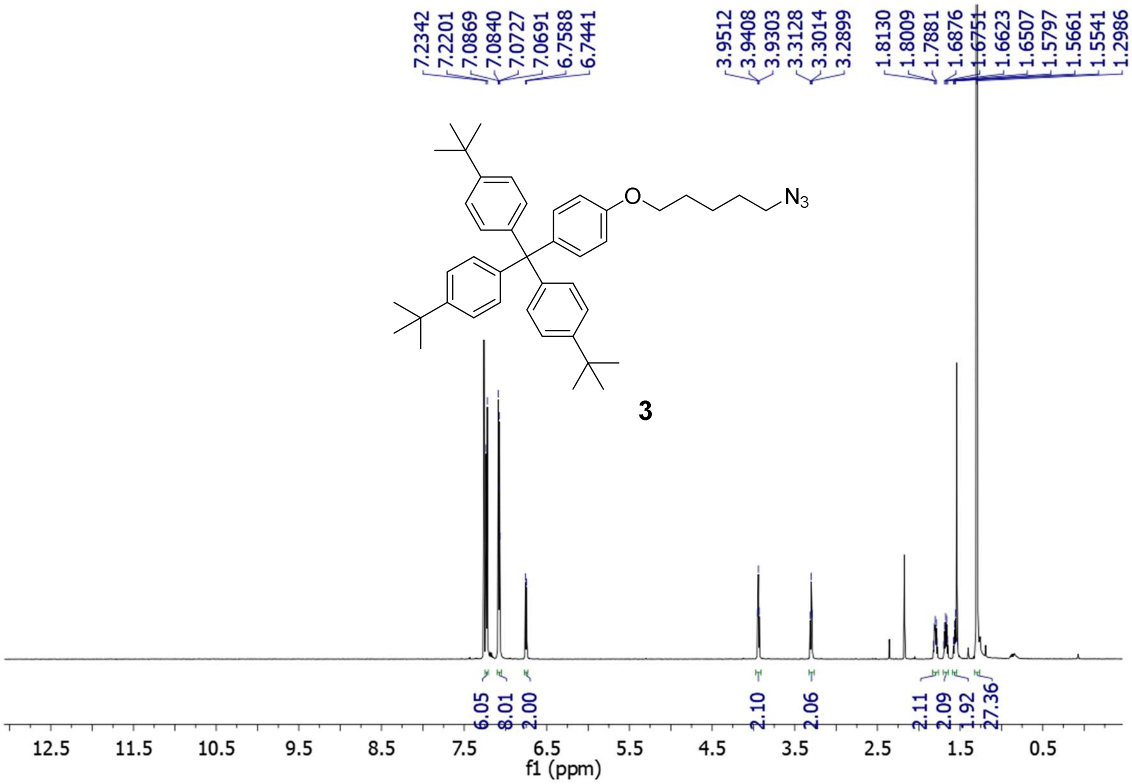

Figure S4: ^{1}H NMR ($CDCl_3$, 600 MHz) spectrum of compound **3**.

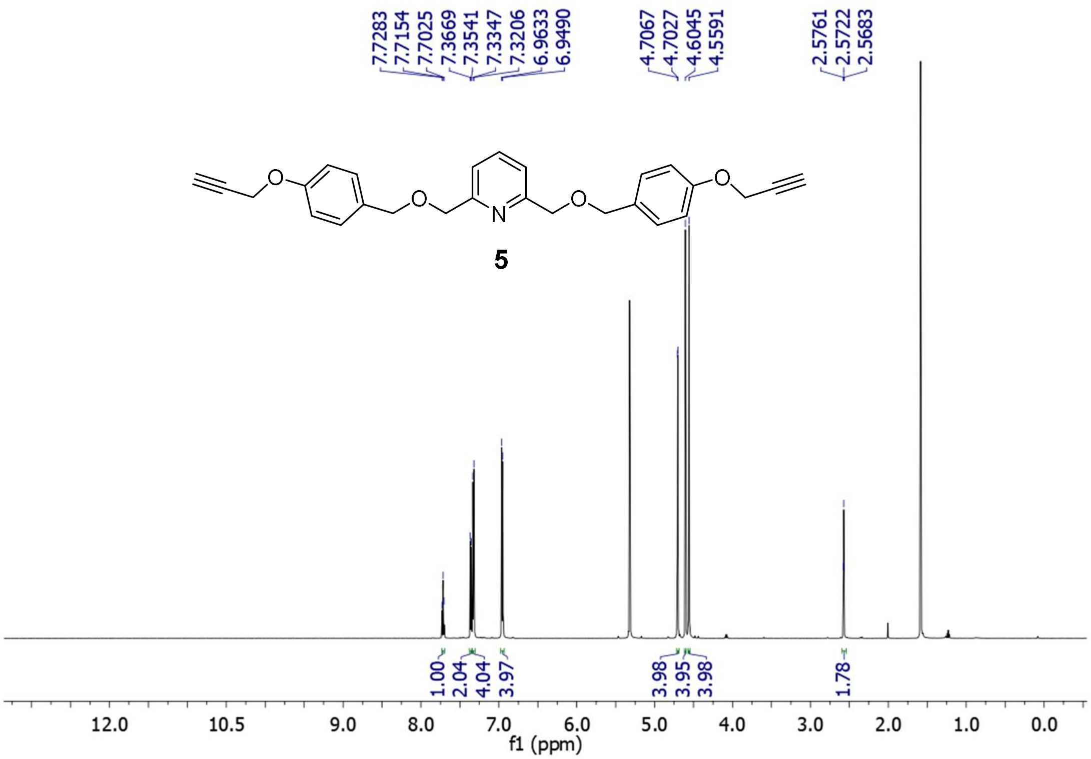

Figure S5: ^{1}H NMR (CD_2Cl_2, 600 MHz) spectrum of compound **5**.

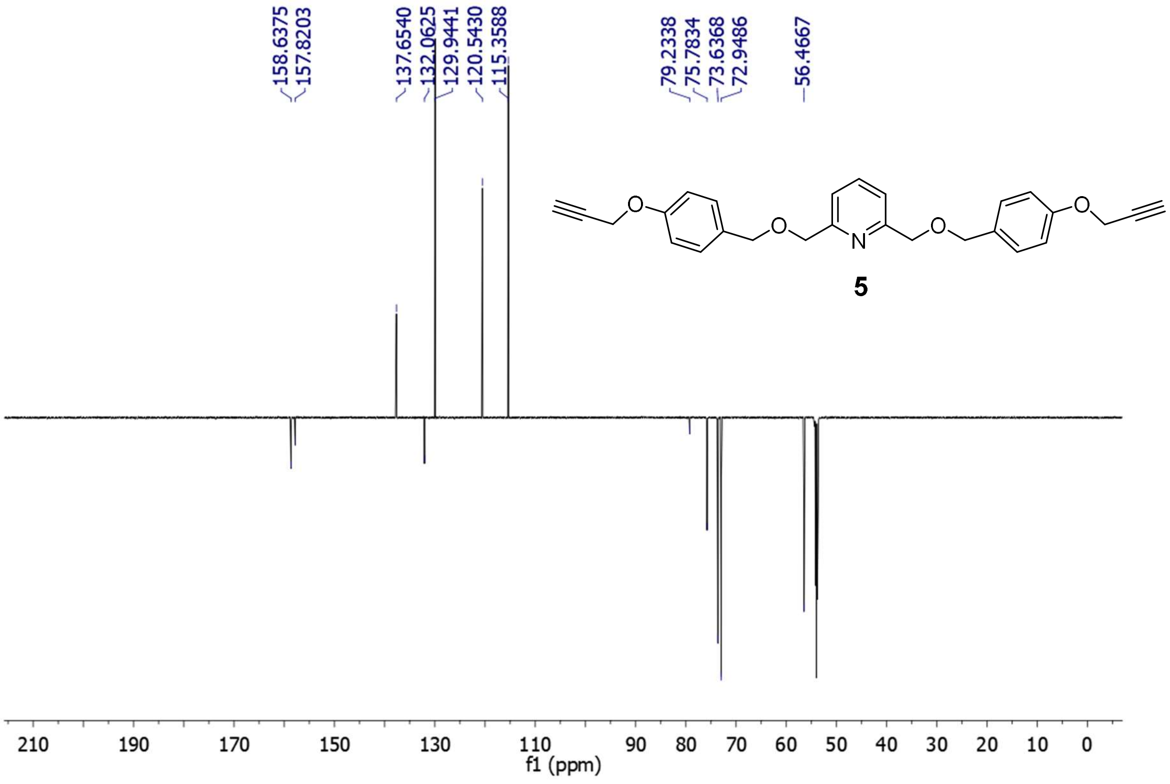

Figure S6: ^{13}C APT NMR (CD_2Cl_2, 150 MHz) spectrum of compound **5**.

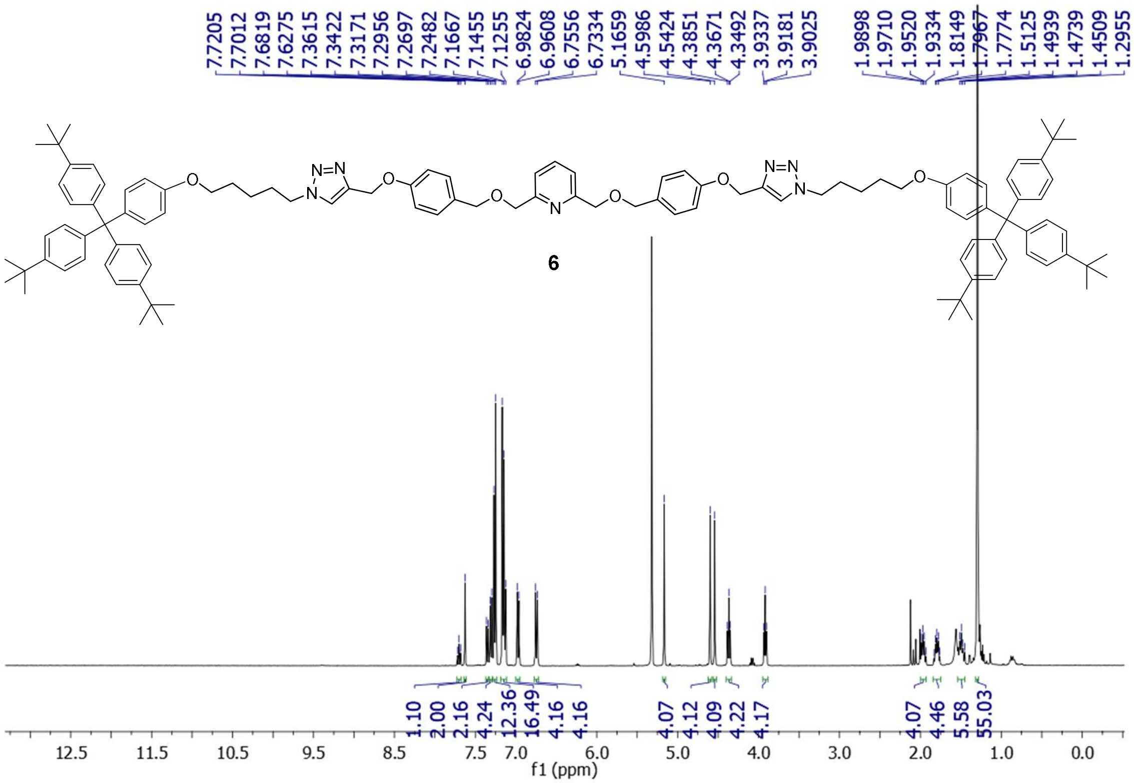

Figure S7: ^{1}H NMR (CD_2Cl_2, 400 MHz) spectrum of compound **6**.

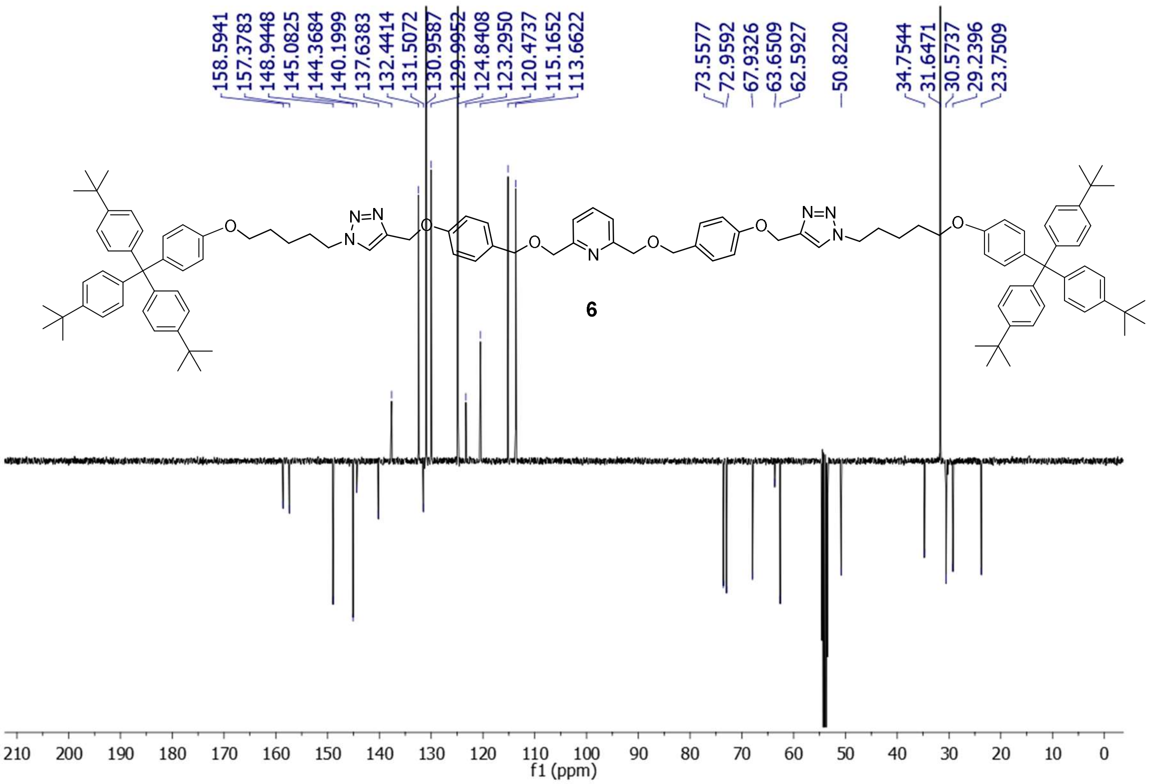

Figure S8: ^{13}C APT NMR (CD_2Cl_2, 100 MHz) spectrum of compound **6**.

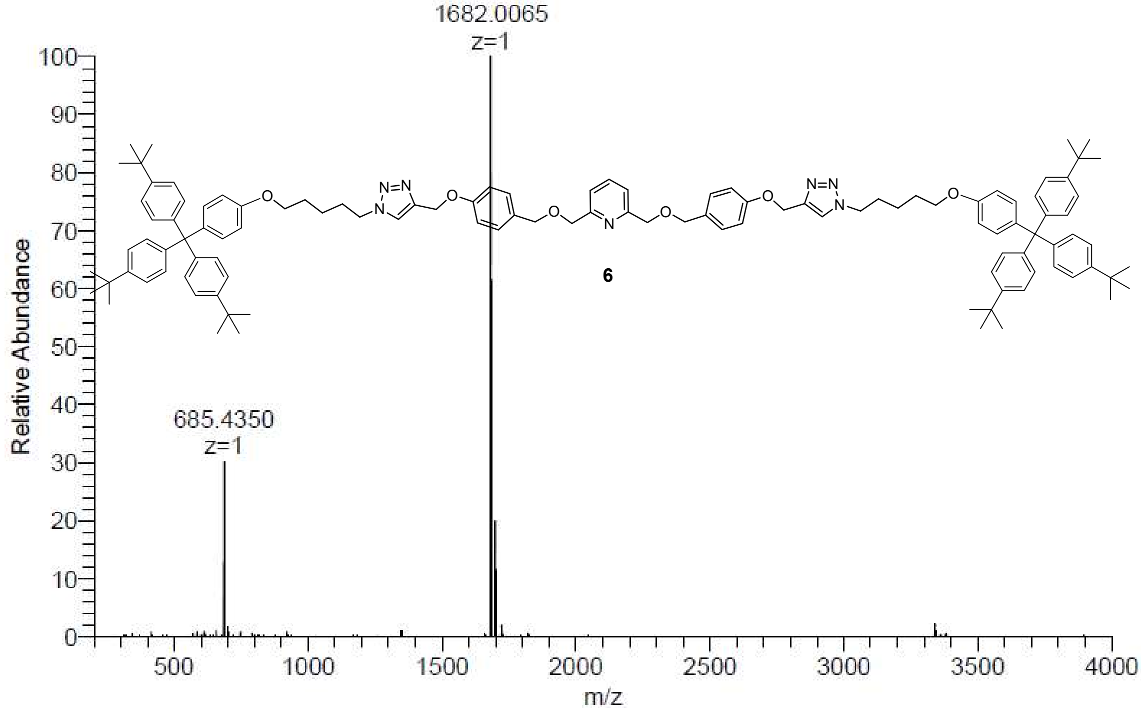

Figure S9: HRESI(+)-MS spectrum of compound **6**.

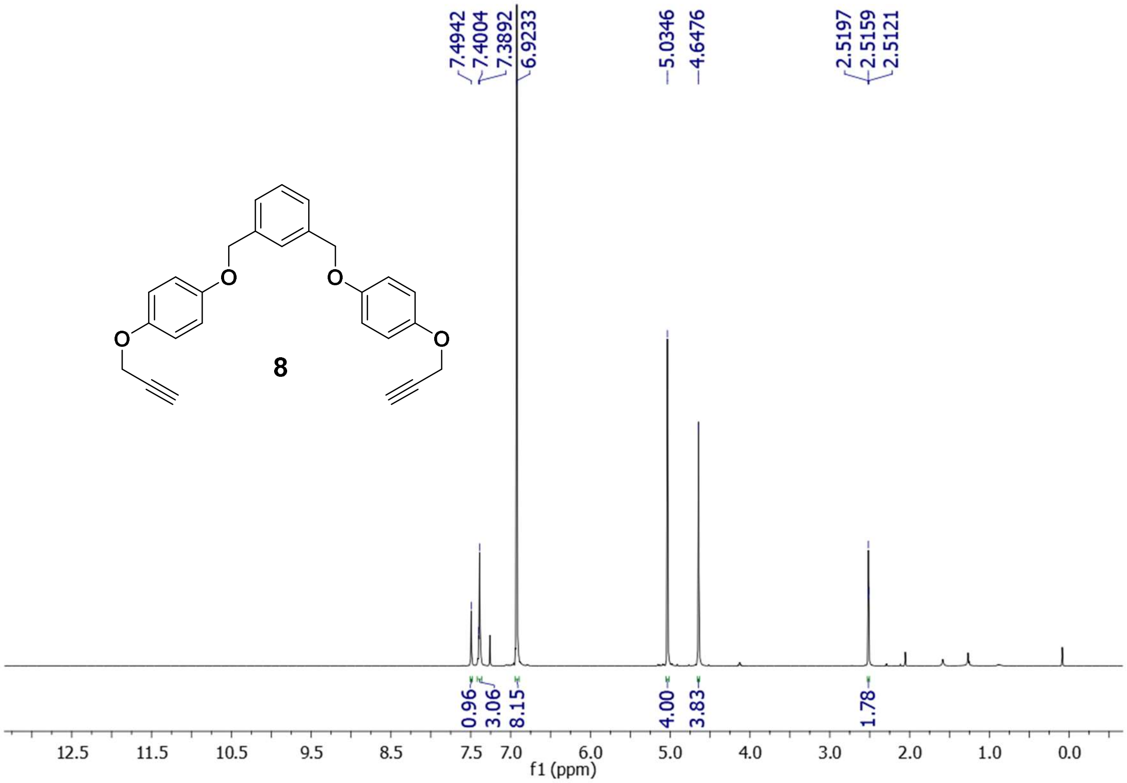

Figure S10: ^{1}H NMR ($CDCl_3$, 600 MHz) spectrum of compound **8**.

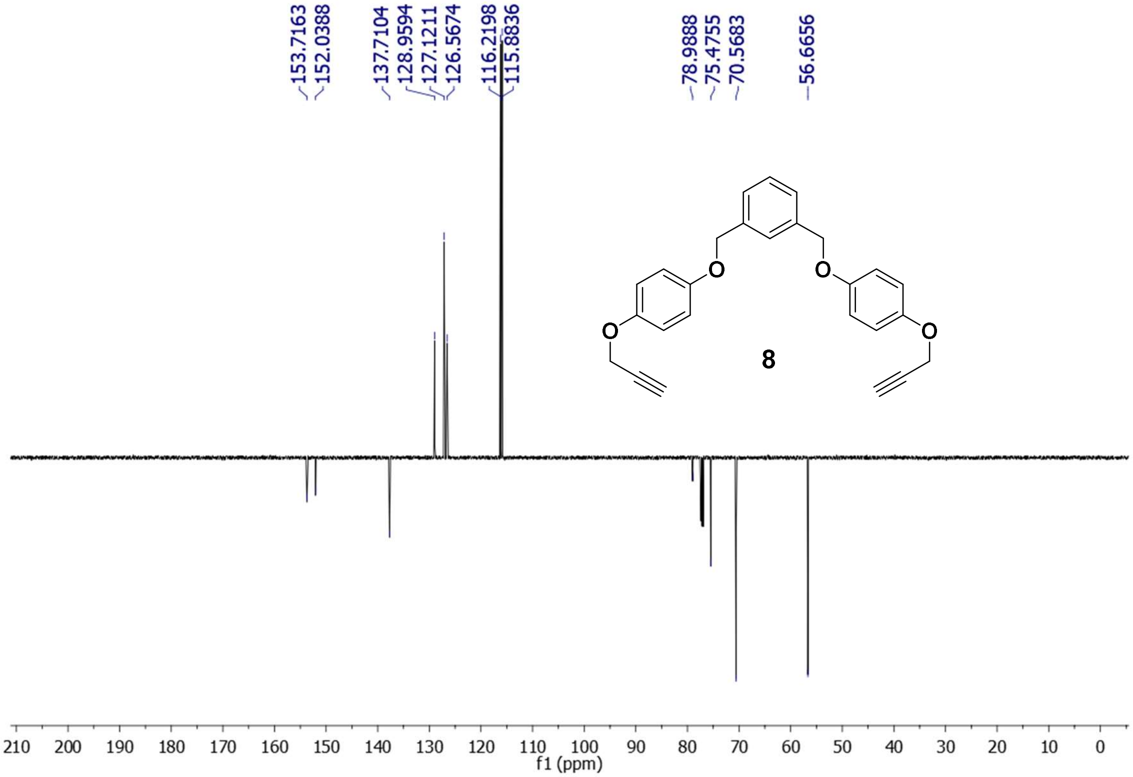

Figure S11: ^{13}C APT NMR ($CDCl_3$, 150 MHz) spectrum of compound **8**.

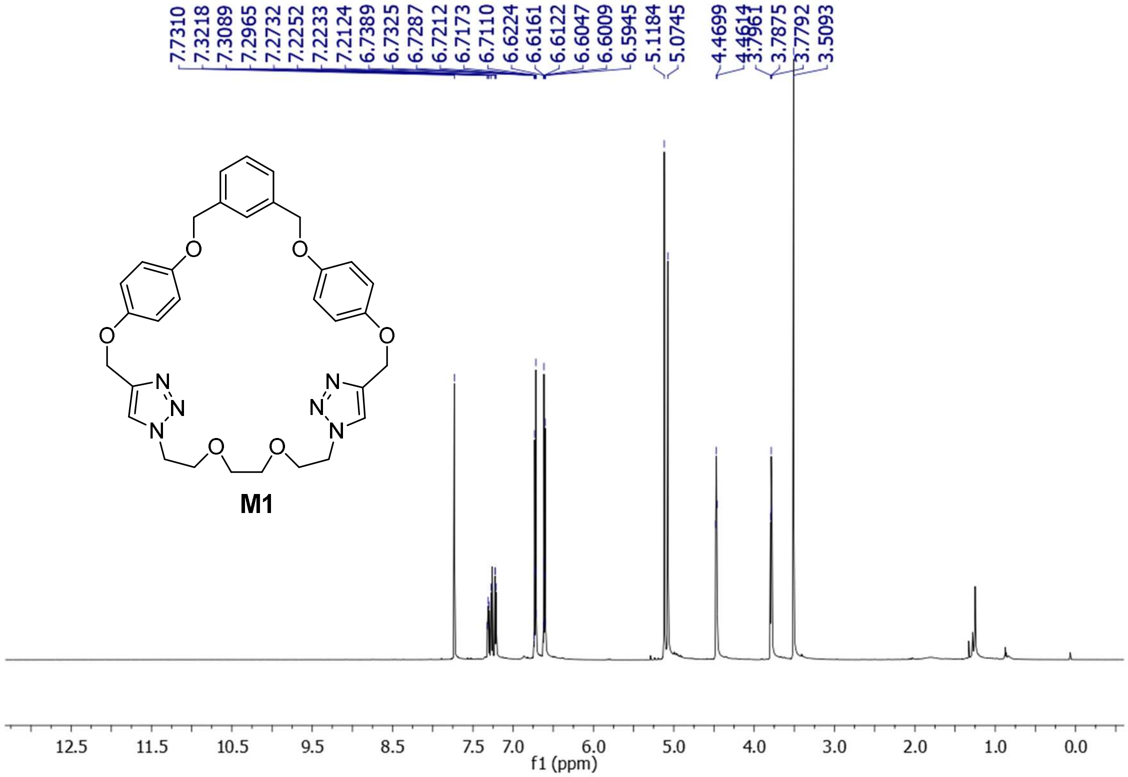

Figure S12: ^{1}H NMR ($CDCl_3$, 600 MHz) spectrum of compound **M1**.

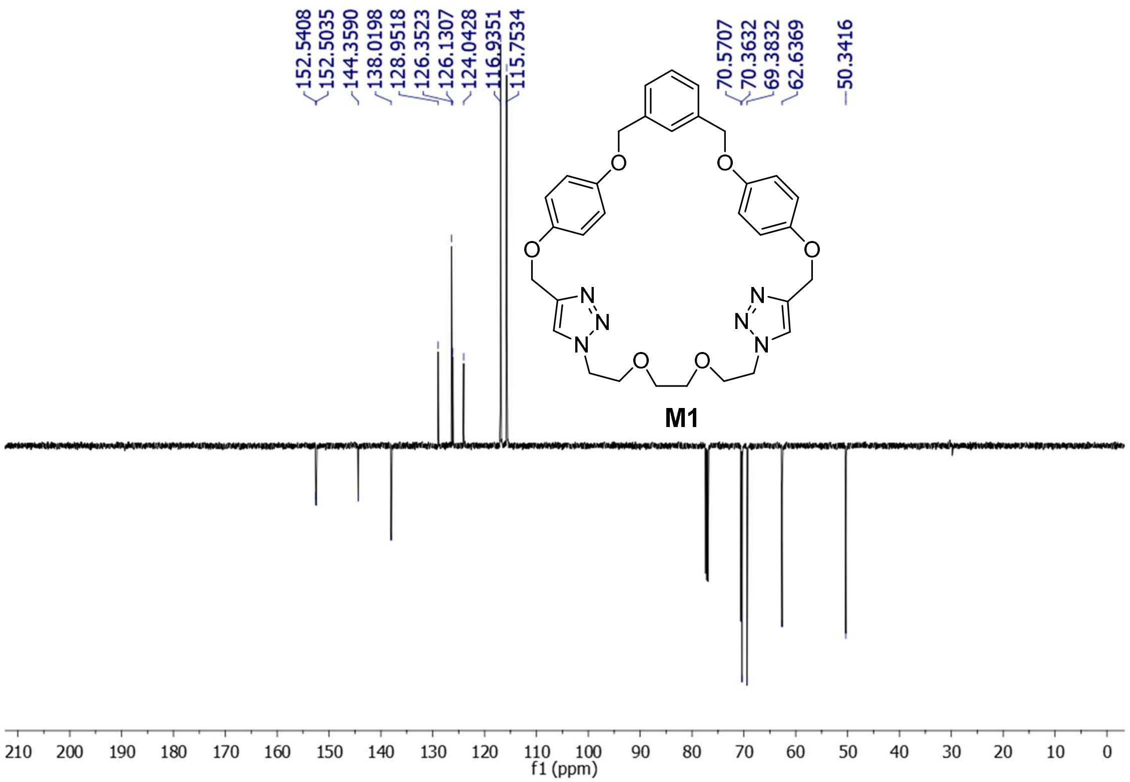

Figure S13: ^{13}C APT NMR ($CDCl_3$, 150 MHz) spectrum of compound **M1**.

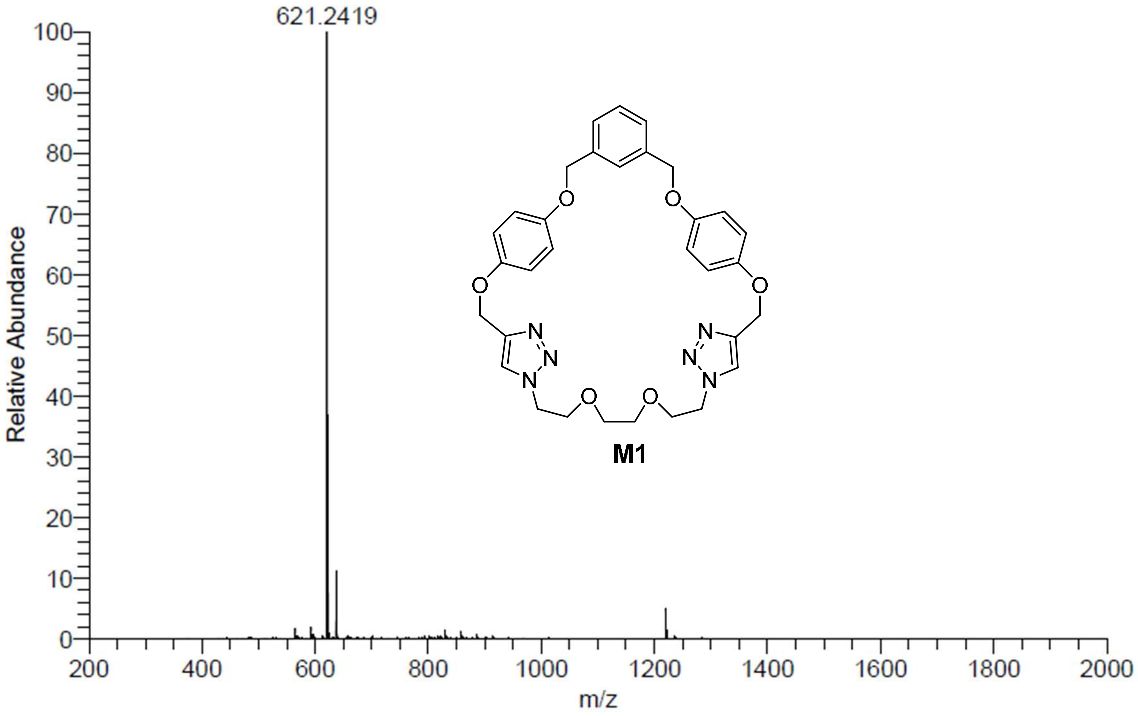

Figure S14: HRESI(+)-MS spectrum of compound **M1**.

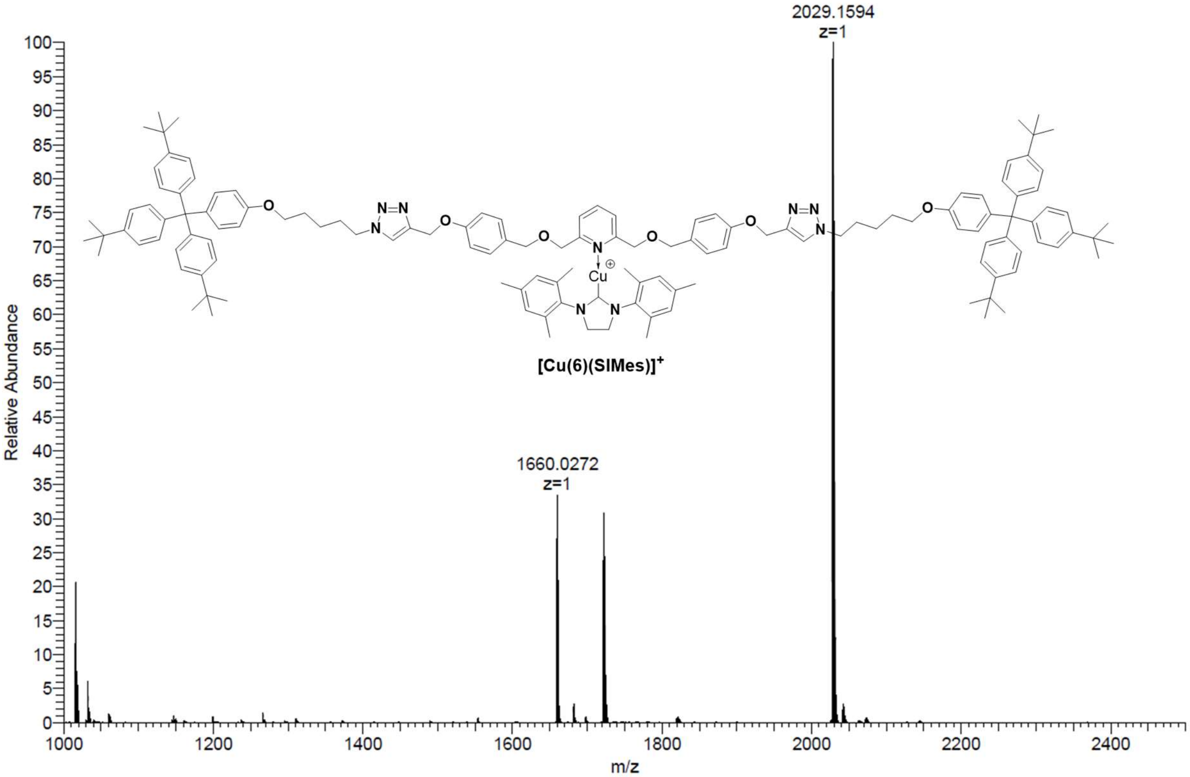

Figure S15: HRMS spectrum of the complex between axle **6** and **CuCl(SIMes)**.

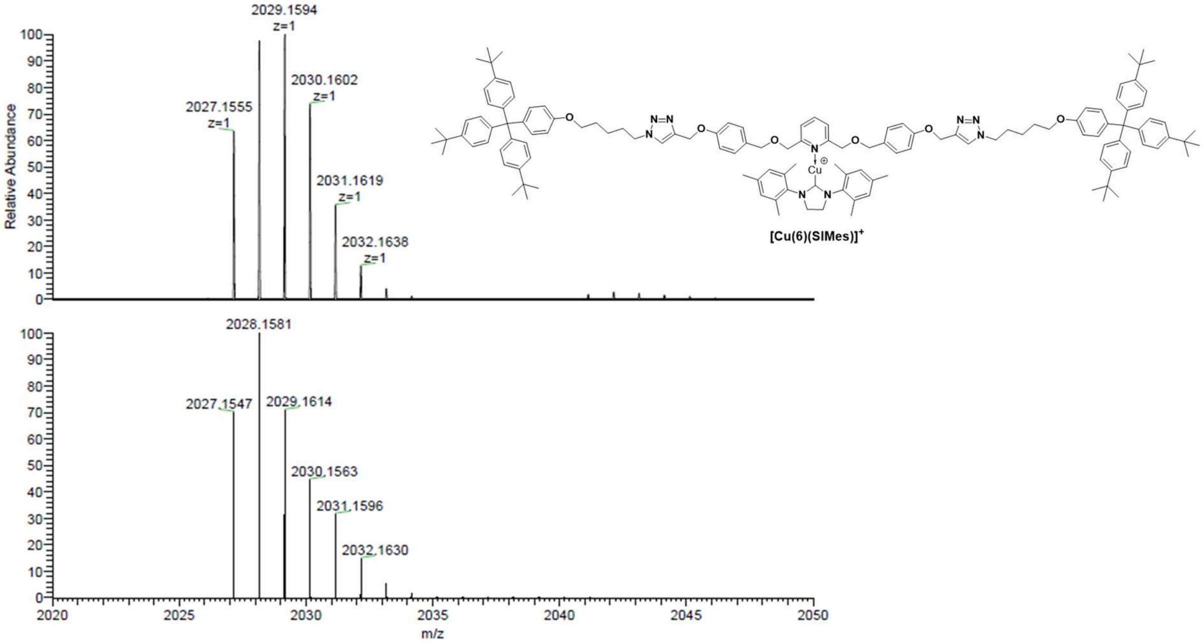

Figure S16: HRMS spectrum of the complex between axle **6** and **CuCl(SIMes**) [experimental (top) and calculated (bottom) isotopic patterns].

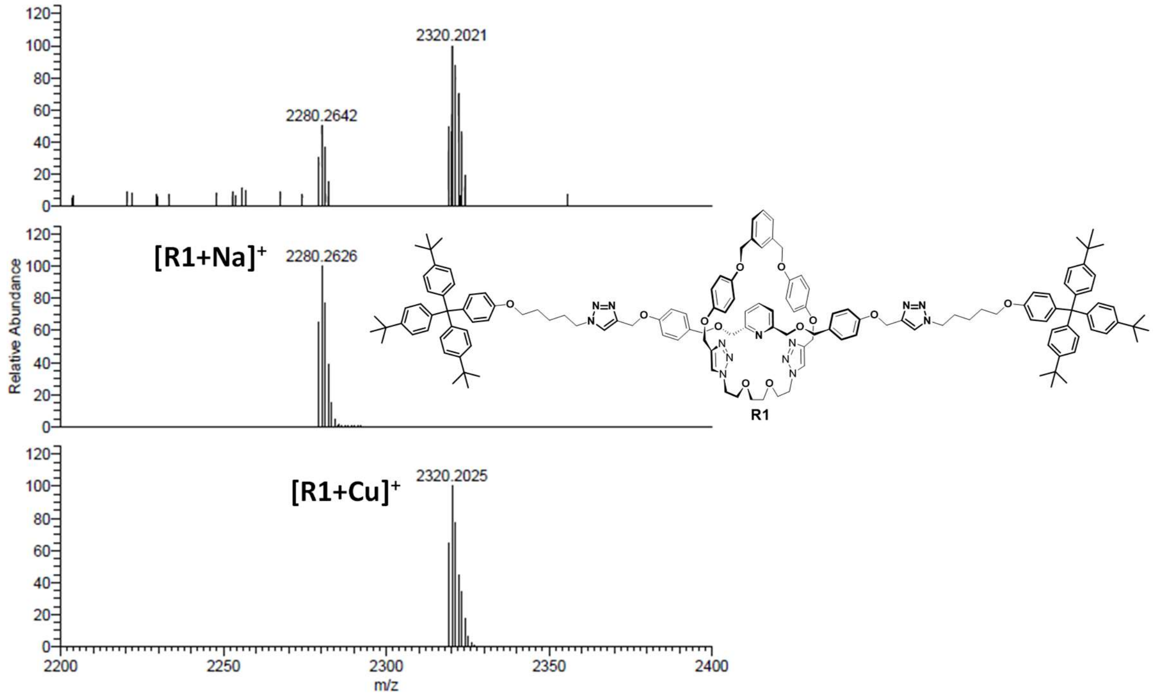

Figure S17: HRESI(+)-MS spectrum of the rotaxane **R1** [experimental (top) and calculated (bottom) isotopic patterns].

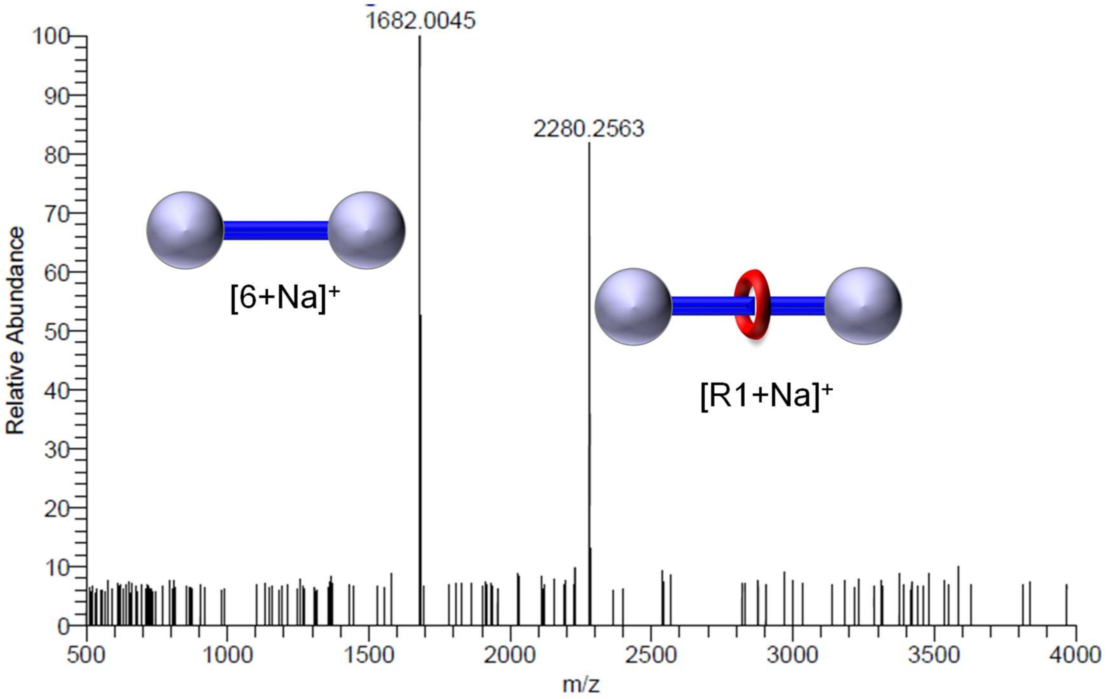

Figure S18: ESI(+)-MS^2 spectrum of rotaxane **R1**.

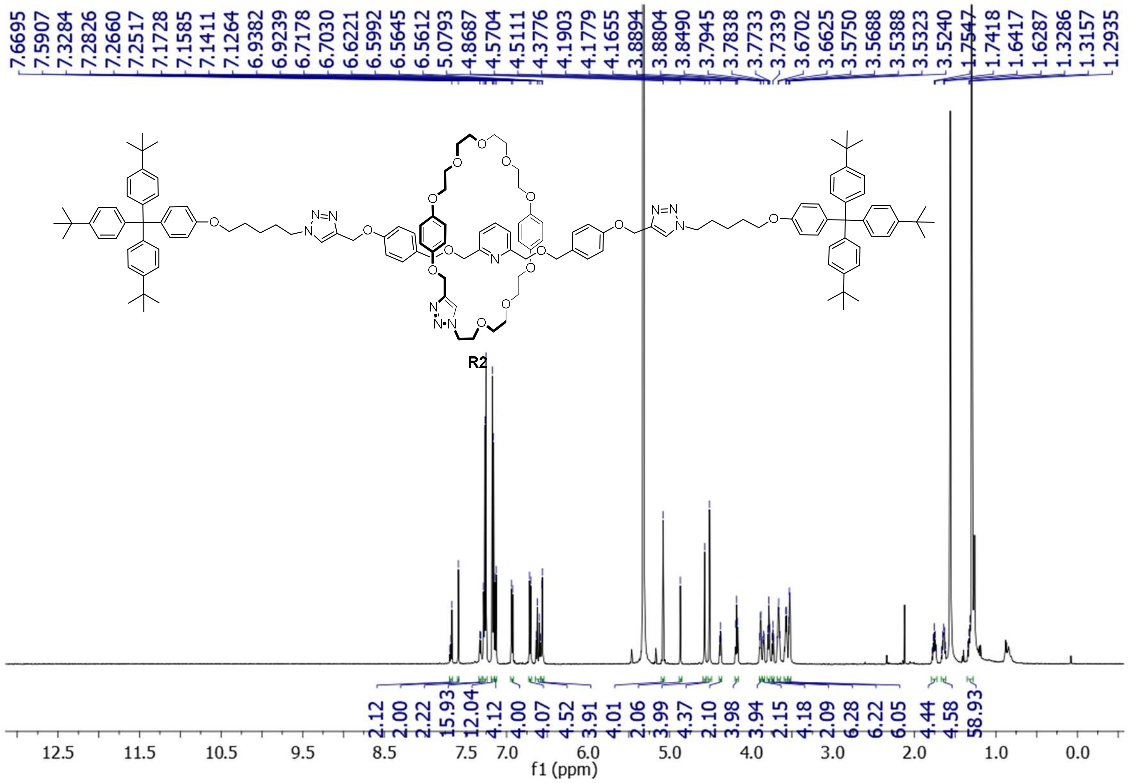

Figure S19: ^{1}H NMR (CD_2Cl_2, 600 MHz) spectrum of compound **R2**.

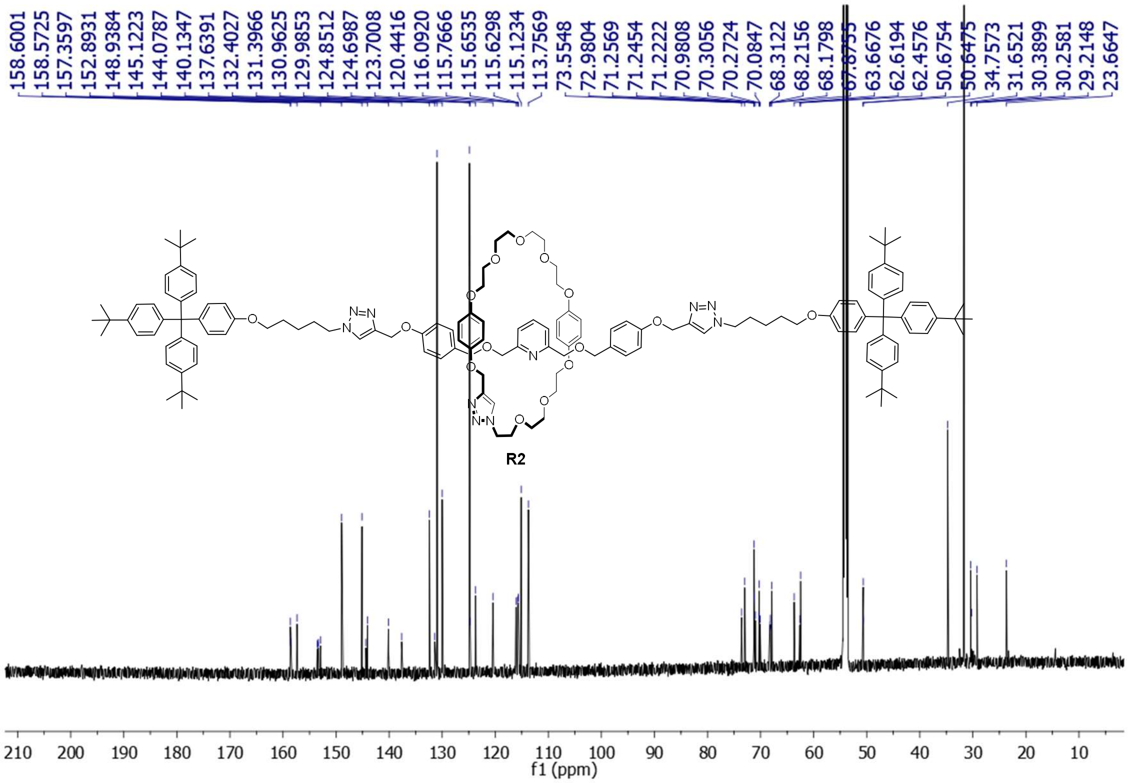

Figure S20: ^{13}C NMR (CD_2Cl_2, 150 MHz) spectrum of compound **R2**.

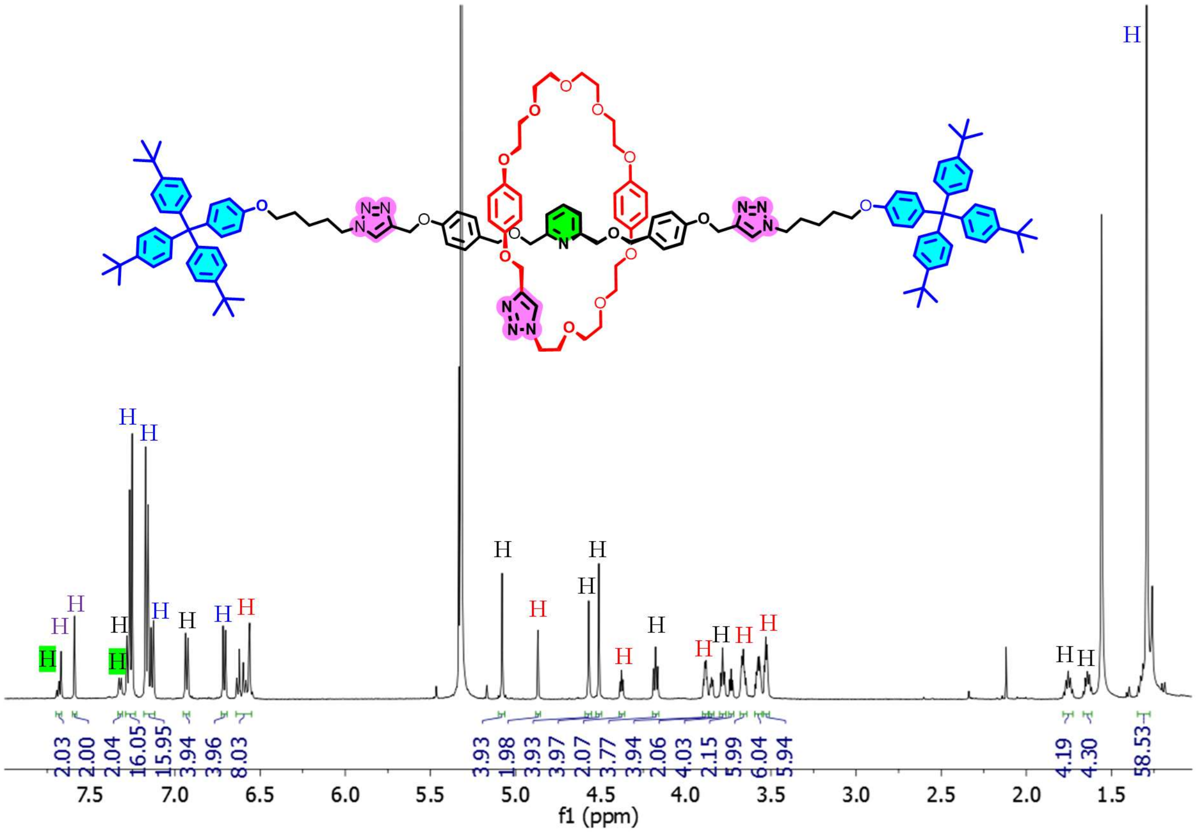

Figure S21: Zoom ^{1}H NMR (CD_2Cl_2, 600 MHz) spectrum of compound **R2**.

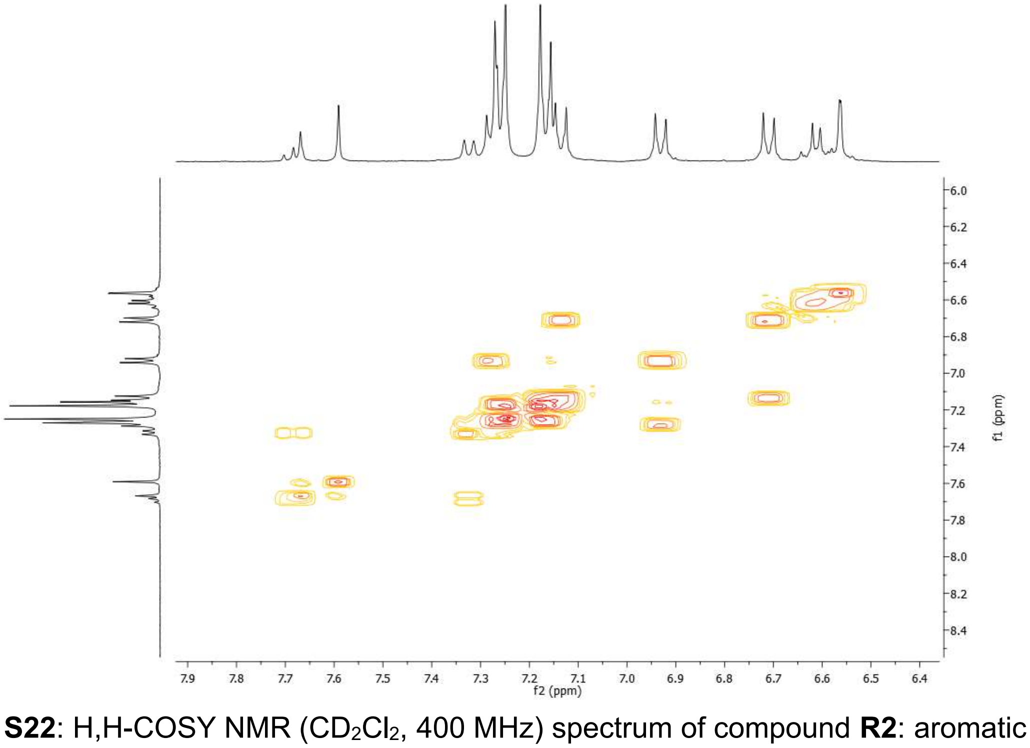

Figure S22: H,H-COSY NMR (CD_2Cl_2, 400 MHz) spectrum of compound **R2**: aromatic region.

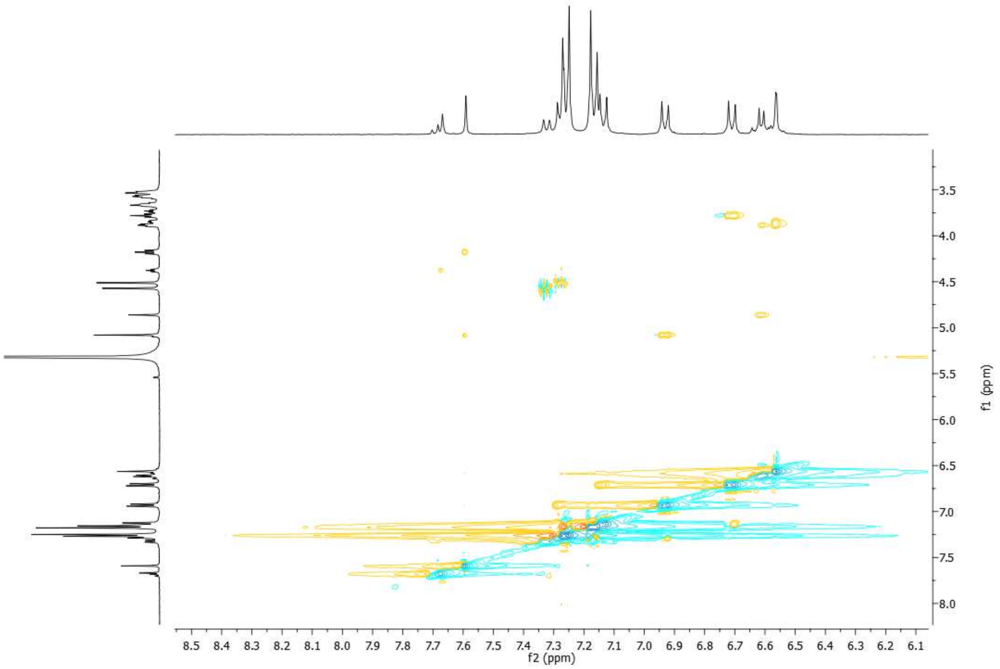

Figure S23: H,H-ROESY NMR (CD_2Cl_2, 400 MHz) spectrum of compound **R2**: aromatic region.

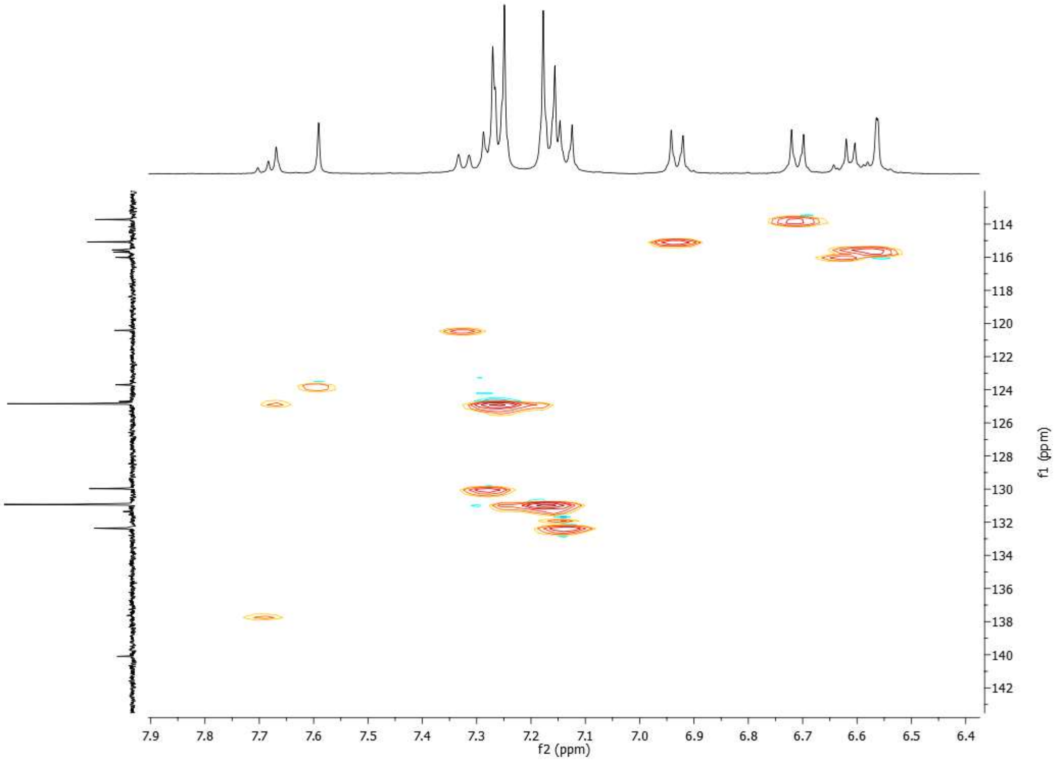

Figure S24: HSQC NMR (CD_2Cl_2, 400 MHz) spectrum of compound **R2**: aromatic region.

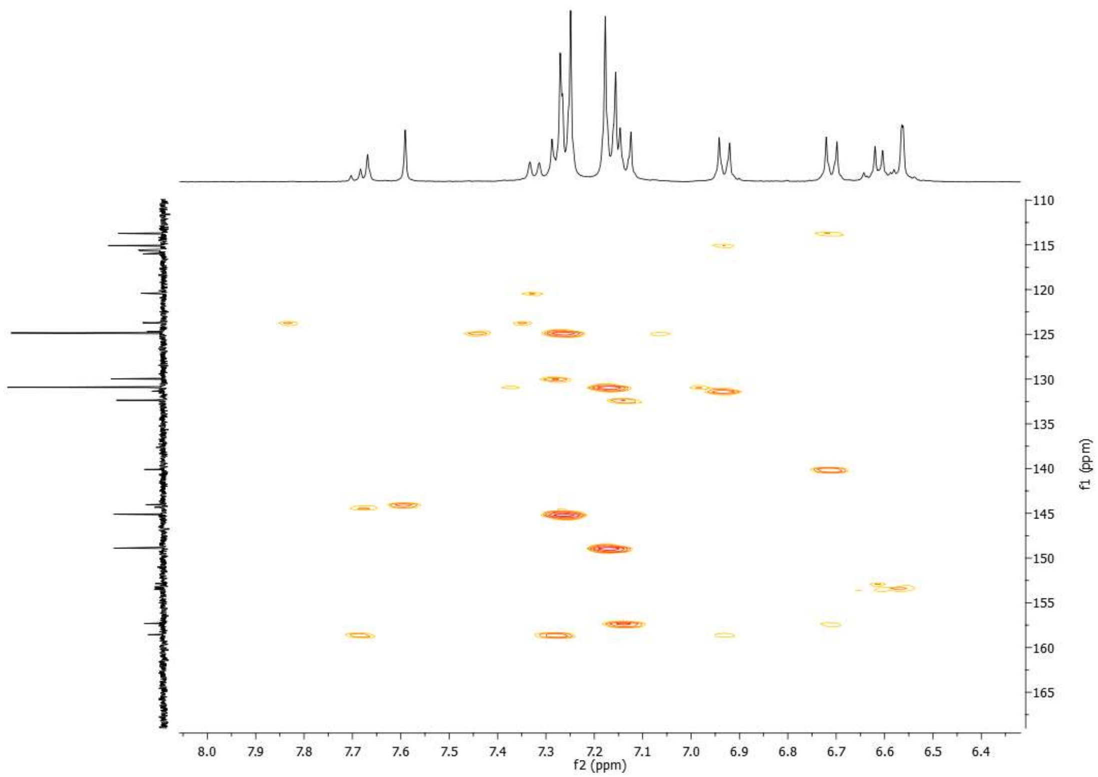

Figure S25: HMBC NMR (CD_2Cl_2, 400 MHz) spectrum of compound **R2**: aromatic region.

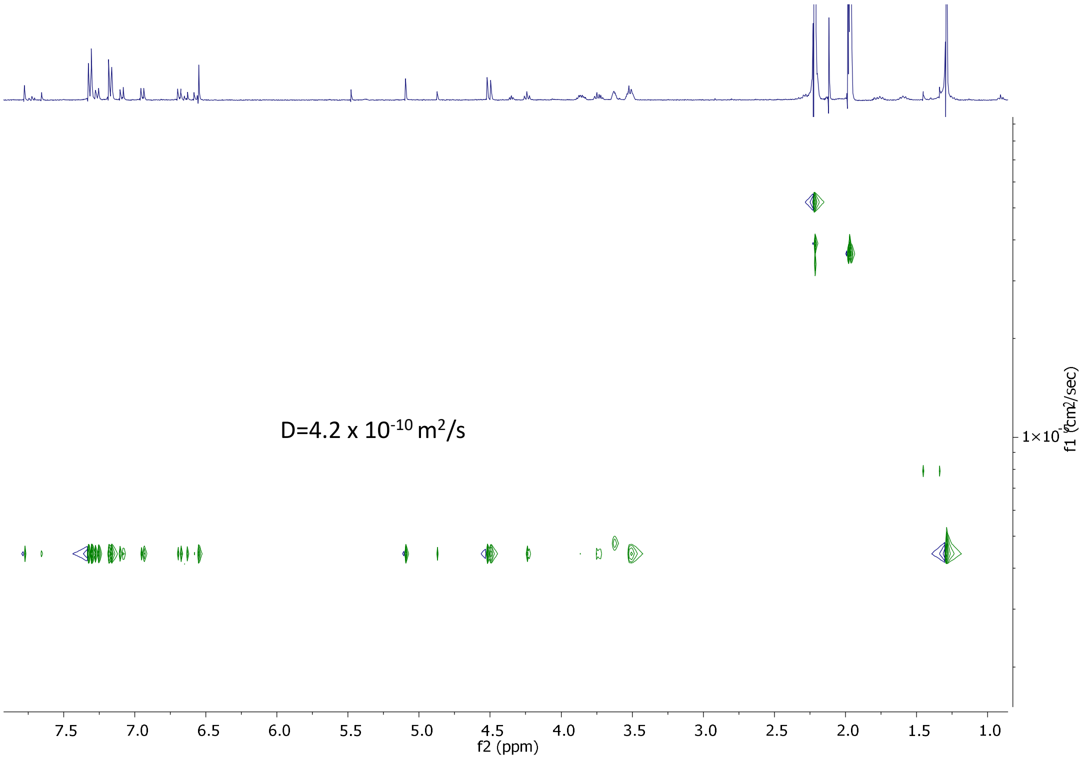

Figure S26: ^{1}H-DOSY NMR (MeCN-d_3, 400 MHz) spectrum of compound **R2**.

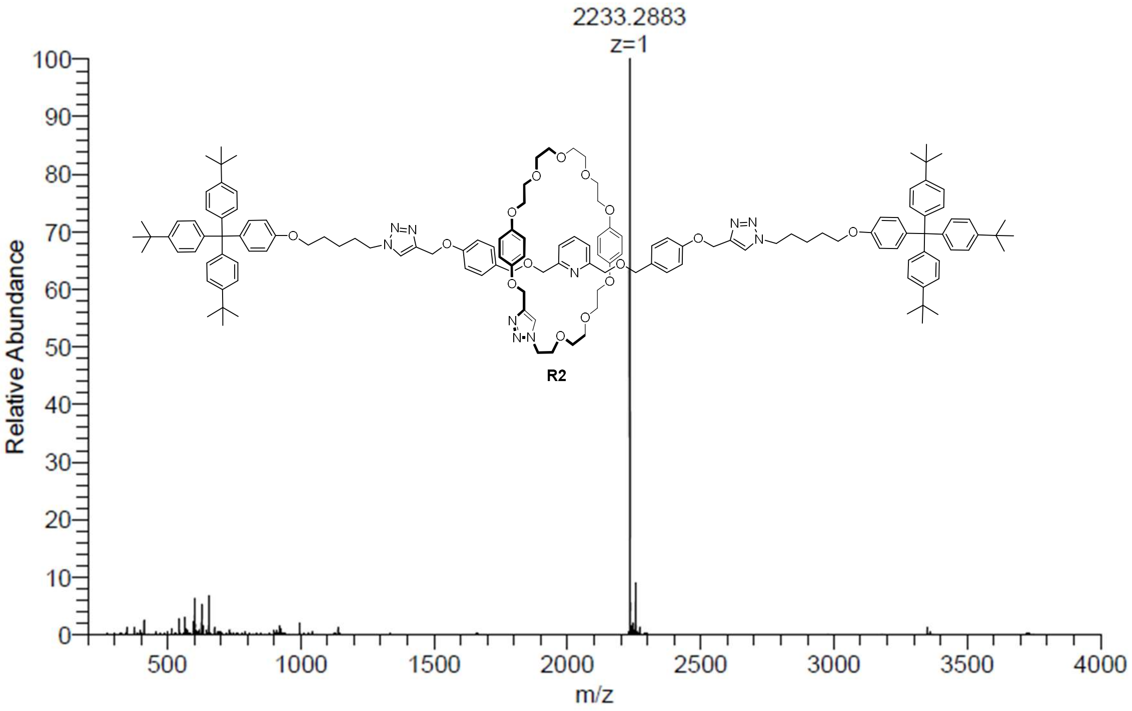

Figure S27: HRESI(+)-MS spectrum of compound **R2**.

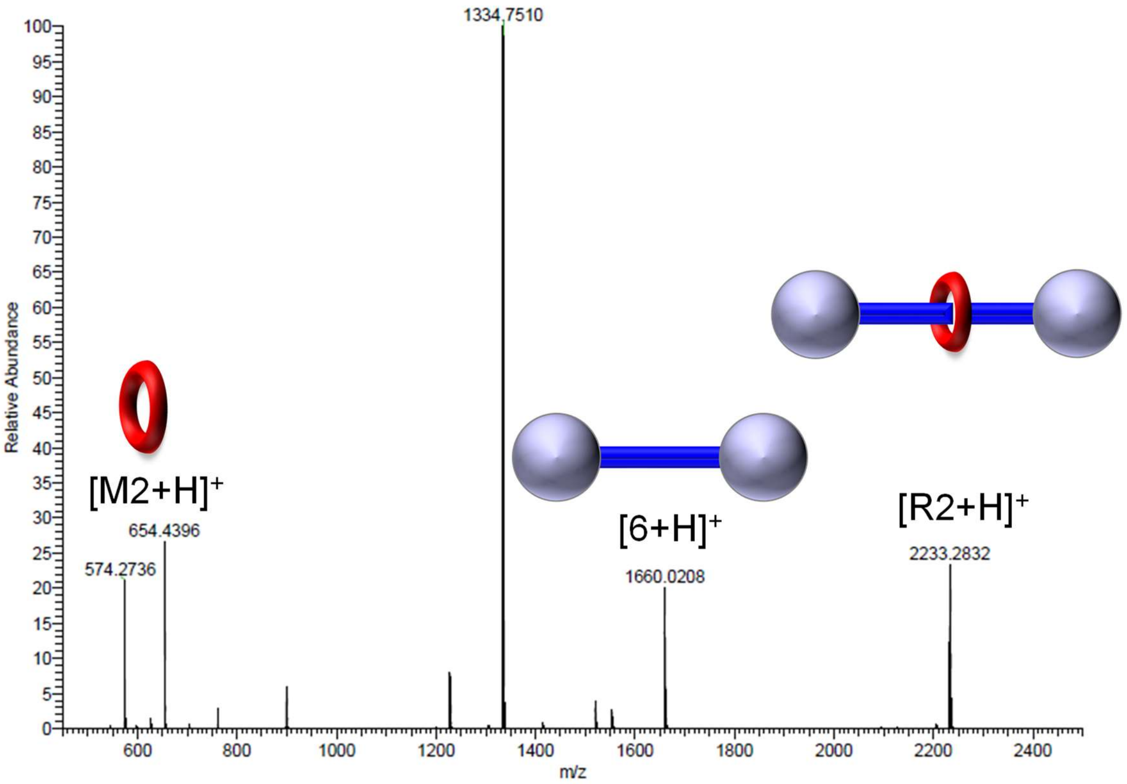

Figure S28: ESI(+)-MS^2 spectrum of rotaxane **R2**.
